# Cell-based sialoglycan arrays for directly comparing influenza A virus receptor requirements for binding and infection

**DOI:** 10.1016/j.isci.2025.112549

**Published:** 2025-05-03

**Authors:** Mengying Liu, Xuesheng Wu, Martijn D.B. van de Garde, Yoshiki Narimatsu, Frank J.M. van Kuppeveld, Henrik Clausen, Cornelis A.M. de Haan, Erik de Vries

**Affiliations:** 1 Virology Section, Division of Infectious Diseases and Immunology, Department of Biomolecular Health Sciences, Faculty of Veterinary Medicine, Utrecht University, Utrecht, the Netherlands; 2 Copenhagen Center for Glycomics, Department of Cellular and Molecular Medicine, Faculty of Health Sciences, University of Copenhagen, Blegdamsvej 3, Copenhagen, Denmark; 3 GlycoDisplay ApS, Blegdamsvej 3, Copenhagen, Denmark

**Keywords:** Biological sciences, Microbiology, Natural sciences, Virology

## Abstract

Influenza A viruses multivalently engages sialoglycan attachment factors. Synthetic glycan arrays provide meticulous insight into primary binding specificity but do not capture dynamic post-binding virus-receptor interactions leading to cell entry. Establishing an HEK293 cell-based array of genetically dissected sialoglycan assemblies enabled screening of the complete interaction cascade from binding to infection, at physiologically relevant low virus doses. Screening forty years of H3N2 receptor binding evolution showed that besides N-glycans, deemed as principal receptors for primary attachment, specific O-glycans or glycosphingolipids independently supported all steps from primary binding to entry. For all three glycoconjugate classes, receptor preferences gradually evolved toward utilization of human-type α2-6-linked sialic acid receptors, followed by regaining use of avian-type α2-3-linked receptors after 1995. The screen identified a lack of quantitative correlation between binding and infection efficiency, suggesting specific receptor requirements beyond attachment. Virus-glycan interactions and other sialoglycan-dependent interactions with cells can be functionally analyzed using this system.

## Highlights

• Cell-based glycan arrays support direct comparison of virus binding and infection
• All three glycoconjugate classes efficiently support influenza A virus infection
• Use of O-glycan and glycolipid receptors by H3N2 gradually declines after 1968

## Graphical abstract

**Figure:**
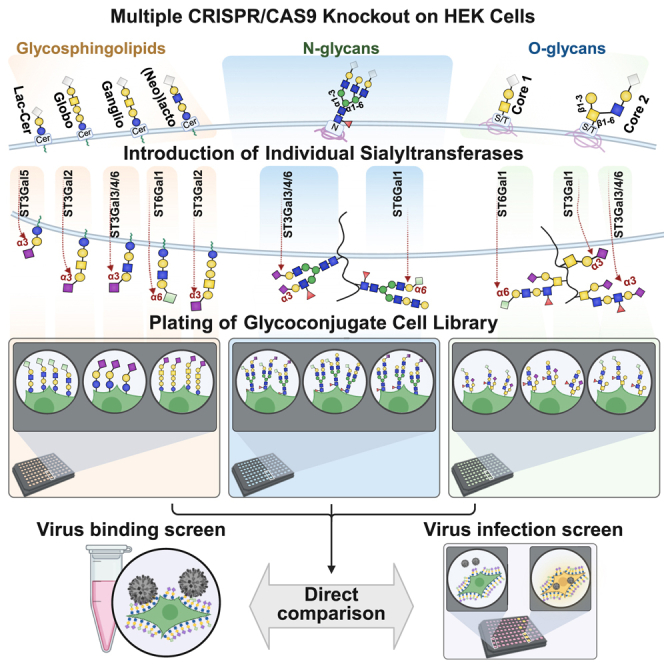

## Introduction

Influenza A viruses (IAV) of birds and mammals bind sialic acid (Sia) receptors on cells, followed by cell entry via endocytosis. Sias constitute a group of monosaccharides capping glycan chains attached to proteins and lipids. Structural diversity of the underlaying glycan chains is a major determinant of IAV host and cell-type specificity.^1^^,^^2^ Waterfowl, comprising the natural reservoir of IAV, harbors ∼140 IAV genotypes that are systematically classified (H1N1 etc.) on the basis of their envelope proteins hemagglutinin (HA; 19 subtypes) and neuraminidase (NA; 11 subtypes). Several genotypes regularly infect poultry and mammals but successful establishment in a new host species is rare. It requires, among others, adaptation to the distinct Sia repertoire of a new host by mutation of Sia-binding HA as well as Sia-cleaving NA for efficient binding and entry.^3^^,^^4^

Switching from avian IAV binding preference for α2-3-linked Sias (2-3Sia) to efficient 2-6Sia binding, was identified as being essential for adaptation to the human host receptor repertoire.^5^^,^^6^ Sia-linkage type refers to the α2-3 or α2-6 bond connecting Sias to the penultimate sugar (mostly galactose) and displays host and tissue-specific distribution. 2-3Sias dominate (but coexist with α2-6Sias) at the avian intestinal tract epithelium and 2-6Sias abound at the human upper respiratory tract which grossly matches with preferred infection sites of, respectively, avian and human IAVs.^7^^,^^8^^,^^9^ This absolute dichotomy of a 2-3Sia/2-6Sia binding in avian versus human IAVs has recently been nuanced.^10^^,^^11^^,^^12^ Whereas two amino acid substitutions in HA were sufficient for a full receptor binding specificity shift,^1^^,^^2^ these substitutions did not prevent binding of H3N2 isolates from the 1968 influenza pandemic to both 2-3Sia and 2-6Sia receptors^11^ and transformation into a highly 2-6Sia-specific human IAV was less fast and essential as originally supposed.^13^

Screening of IAV binding preferences often employs glycan arrays displaying a variety of glycans homogeneously coated at high density. Large effects on binding strength by the diverse structures subterminal to the Sia moiety were shown.^14^^,^^15^^,^^16^ However, glycans displaying strong binding are not necessarily the ultimate receptor for a particular IAV strain as the cell surface displays a complex mixture of diverse sialoglycans. IAV particle binding is multivalent by necessity as only multiple HA-Sia interactions can collectively cause high avidity virus-surface interaction. The individual HA-Sia interactions occur with a variety of different glycans displaying a range of weak affinities (K_D_ is ∼1–50 mM^13^). Such a heteromultivalent binding mode enables efficient utilization of a complex cell surface receptor repertoire.^17^

A collective contribution of low and high affinity HA-Sia interactions to virus binding expands the range of relevant IAV receptors.^17^ Glycoconjugate classes like glycosphingolipids (GSLs) or N-glycans and O-glycans attached to proteins contribute to Sia-dependent IAV binding.^18^^,^^19^^,^^20^^,^^21^^,^^22^^,^^23^ Branch length and sub-terminal modifications of N-glycans, the supposed major IAV cell surface receptor, affect IAV binding in a strain-specific way^16^ but the fine-structures of GSLs and O-glycans contributing to infection require further investigation. This also counts for the decoy receptor function of heavily O-glycosylated mucins present in the mucus layers covering all wet lining epithelia.^24^^,^^25^ The correlation between IAV binding strength and infection efficiency is still poorly resolved. This questions the requirements for IAV-receptor interactions in the steps subsequent to primary binding that lead to cell infection. Clearly, methods for screening the receptors involved in these steps need to be developed.

To this end HEK293 cells were genetically modified to establish a cell-based sialoglycan array that was validated by lectin binding and screened for glycan assemblies specifically supporting binding and infection of IAV strains. Practical advantages of HEK293 cells include robust growth rates, easy maintenance as adherent or suspended cells, high and reproducible transfection levels, efficient gene engineering by CRISPR-Cas techniques and wide use for IAV infection. Moreover, HEK293 cells support large yield expression of heterologous proteins, which enables efficient production of specific glycoforms of glycoproteins for use in binding analysis in direct comparison to infection. Being of fetal human embryonic origin, the exact cell type is unknown but the morphology is epithelial and cilia and microvilli are displayed. They can be infected efficiently by a wide range of viruses and infection by IAVs is similar in efficiency and utilization of entry pathways as for the human lung epithelial cell line A549^26^ as well as many other immortalized cell lines. HEK293 cell lines displaying either of the three major glycoconjugate classes^11^ were depleted from β-galactoside-sialyltransferases (STs) by CRISPR-Cas gene knock-out. Sialoglycan expression on individual glycoconjugates was reconstituted by systematic reintroduction of individual STs and tested for IAV binding and infection. Avian H5N1, pandemic H1N1, and H3N2 strains isolated from 1968 to 2009 displayed differences in receptor utilization and remarkable evolution of H3N2 receptor preferences. Virus binding to cell arrays or cell array-derived recombinant glycoproteins enabled an integrated approach for comparing binding and infection supported by specific glycan assemblies displayed in the natural context of the cell membrane. The moderate correlation between binding and infection emphasizes a role for specific virus-receptor interactions subsequent to primary binding.

## Results

### Human IAV infection loses and regains support of O-glycans and glycolipids over time

**Figure 1:**
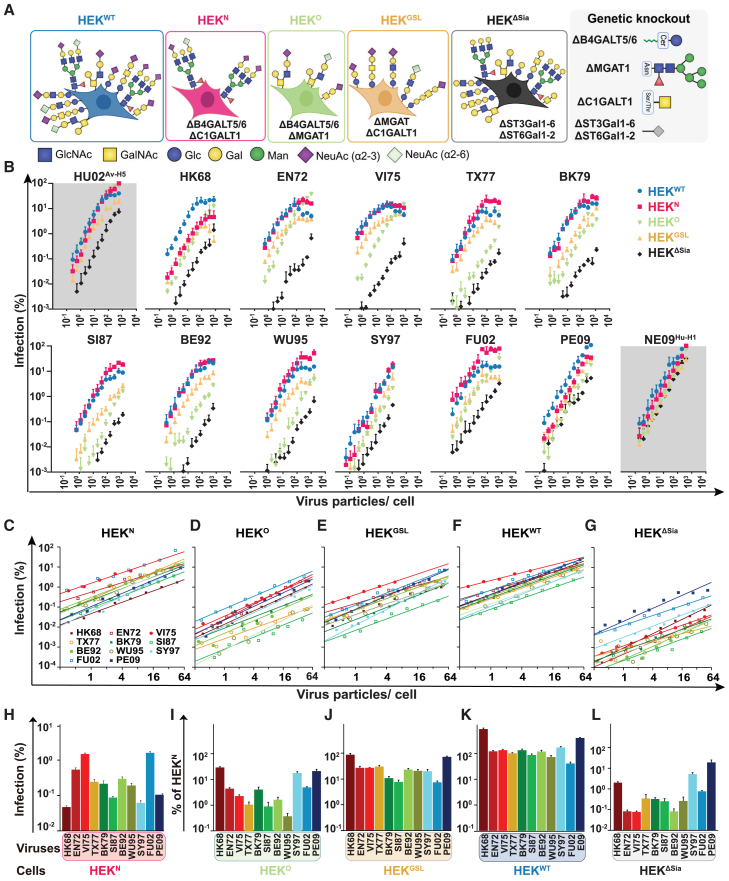
Evolution of receptor utilization for infection by human H3N2 strains (A) Schematic illustration of CRISPR-Cas9-directed truncation of glycoconjugates in HEK^WT^ cells. Combinations of glycosyltransferase genes are knocked out in HEK^WT^ cell lines expressing a single glycoconjugate class at full complexity and truncated core structures of the other two classes.^10^ (B) Efficiency of IAV infection into glycoconjugate-specific cells was quantified by viral RNA polymerase-dependent expression of Gaussia luciferase from a reporter construct. Y axis displays infection relative to the highest observed infection level in HEK^WT^ cells (PE09 at highest virus dose). X axis displays virus dose in number of particles/cell. Note that a dose of ∼100 particles/cell (with considerable strain-to-strain variation) compares to a multiplicity of infection of 1 as determined by titration on MDCKII cells.^12^ Infection by human H3N2 isolates from 1968 (HK68) to 2009 (PE09), avian H5N1 (HU02^Av−H5^) and pandemic H1N1 (NE09^Hu−H1^). (C–G) Fitted curves (y = *a*x^*b*^) for data from (B) in the 0.25 to 64 virus particles/cell range (statistics in Table S1) assembled per cell line. Axes as in (B). (H) Infection efficiency into HEK^N^ cells at a virus dose of 1 particle/cell (calculated from (C)). Y axis as in (B). (I–L) Infection efficiency into the other cell lines (HEK^O^, HEK^GSL^, HEK^WT^, and HEK^ΔSia^) relative for each virus to its infection efficiency into HEK^N^ cells (from (H)). Data are represented as mean ± SEM.

Support of IAV infection by single individual glycoconjugate classes was investigated by infection of genetically engineered (see Figure 1A) HEK293 cells (HEK^WT^) expressing Sia on only N-glycans (HEK^N^), only O-glycans (HEK^O^) or only glycosphingolipids (HEK^GSL^)^11^ with serial dilutions of avian H5N1 (HU02^Av−H5^) pandemic H1N1 and H3N2 (NE09^Hu−H1^ and HK68^Hu−H3^) and ten seasonal H3N2 strains isolated between 1972 and 2009. Results were compared to infection of HEK^WT^ cells and Sia-independent infection of HEK^ΔSia^ cells lacking all β-galactoside α2-6 and α2-3 sialyltransferase (STs).^27^ Virus dose was based on particle number to prevent strain-specific biases associated with hemagglutination titers or infectious titers. Avian (HU02^Av−H5^) and early pandemic strains (NE09^Hu−H1^ and HK68^Hu−H3^) were efficiently supported by each sialoglycoconjugate class (Figure 1B). Antigenically drifted seasonal H3N2 strains efficiently used N-glycans receptors whereas infection efficiency of HEK^O^ cells and, to a lesser extent, HEK^GSL^ cells gradually decreased over time. After 1995 (WU95^Hu−H3^), infection efficiency supported by O-glycans or GSLs increased again. Infection by the 2009 isolate PE09^Hu−H3^ was, similar to avian and early pandemic strains, efficiently supported by all three glycoconjugate classes. Notably, maximal receptor selectivity occurred at low infectious doses with curves converging to saturating infection levels at high virus dose.

Data points were fitted with high confidence (Figures 1C–1G; statistics in Table S1) to a power function (y = *a*x^*b*^; x is infection dose in particles/cell, y is infection level and a is infection efficiency at a dose of 1 virus particle/cell; b relates to cooperative effects, when b is 1 there is no cooperativity and infection is directly proportional to virus dose). Infection efficiency of HEK^O^ and HEK^ΔSia^ cells differed up to two orders of magnitude between H3N2 strains. Infection supported by N-glycans (HEK^N^ cells; Figure 1H), considered as the prime IAV receptor class, was used for normalization of infection efficiency of the other cell lines (Figures 1I–1L). For 9 H3N2 strains, N-glycans supported infection to levels as for HEK^WT^ cells, whereas pandemic HK68^Hu−H3^ and PE09^Hu−H3^ displaying 9-fold and 4-fold higher infection of HEK^WT^ cells. In comparison to infection levels observed in HEK^WT^ cells (Figure 1K), infection of HK68^Hu−H3^ was efficiently supported by O-glycans or GSLs (Figures 1I and 1J). After 1968, O-glycan-dependent infection efficiency gradually decreased, with WU95^Hu−H3^ being ∼270-fold less infectious to HEK^O^ cells than to HEK^N^ cells (Figure 1I). After 1995 the trend is reversed with PE09^Hu−H3^ displaying only ∼5-fold less efficient O-glycan-dependent infection in comparison to HEK^N^ cells. Utilization of GSL receptors evolved by a similar trend (Figure 1J), but was maximally (for SI87^Hu−H3^) only ∼13-fold less efficient as for N-glycans. We observed variable levels of Sia-independent infection into HEK^ΔSia^ cells, for which candidate receptors like phosphorylated glycans,^28^ specific protein receptors^29^ or cell surface lectins^30^ binding to HA and NA glycoproteins have previously been proposed. Remarkably, H3N2 infection efficiency of HEK^ΔSia^ cells decreased after 1968 but became much more efficient again after 1997 for unknown reasons. Of caution, ST6GalNAc transferases were not knocked-out in our HEK^ΔSia^ cells, although the complete loss of sialylation in the HEK^ΔSia^ cells was confirmed by a metabolic labeling assay with alkyne-tagged sialic acid.^27^ ST6GalNAc1, 4, 5, and 6 are hardly expressed in HEK293 cells. ST6GalNAc2 and 3 sialylate the GalNAc residue present in core 1 O-glycans (Galβ1-3GalNAcα1-*O*-Ser/Thr) but overexpression of ST6GalNAc2 in HEK^ΔSia^ cells did not increase IAV infection (Figure S1).

In conclusion, each glycoconjugate class efficiently supports pandemic H3N2 (HK68^Hu−H3^), pH1N1 (NE09^Hu−H1^), and avian H5N1 (HU02^Av−H5^) infection. Evolution of seasonal H3N2 strains resulted in preferential utilization of N-glycan receptors, but this was reversed after 1995.

### Generation of a cell-based glycan array displaying specific sialoglycoconjugates

**Figure 2:**
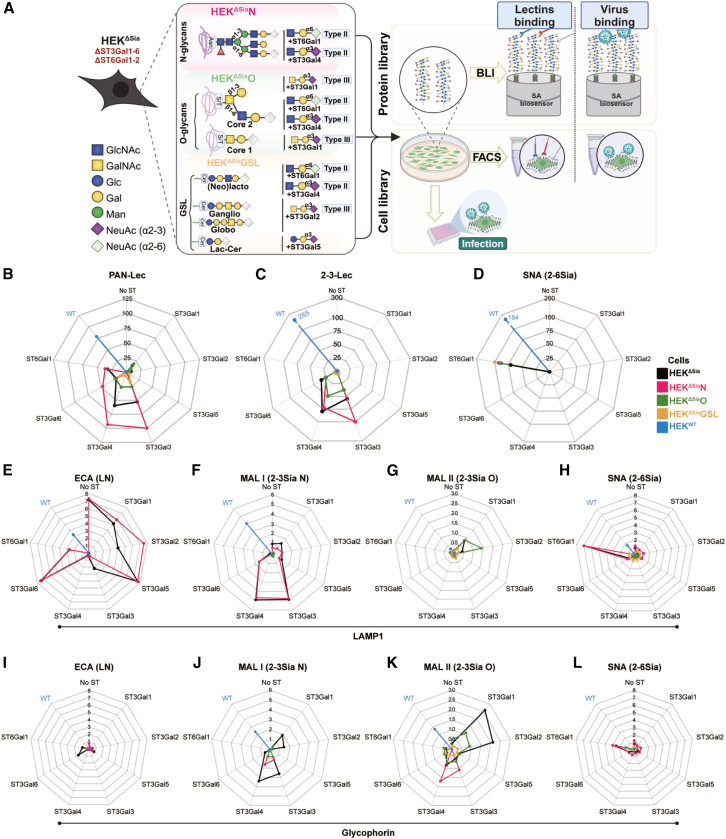
Lectin profiling of sialoglycans installed by transfection of individual specific STs on glycoconjugate-specific cells devoid of endogenous STs (A) Schematic illustration of CRISPR/Cas9-directed truncation of specific glycoconjugate classes in HEK^ΔSia^ cells to produce sialyltransferase-depleted cells expressing only N-glycans (HEK^ΔSia^N), only O-glycans (HEK^ΔSia^O) or only GSLs (HEK^ΔSia^GSL). A sialoglycan-specific cell array is produced by transient introduction of a single ST including ST3Gal1 to ST3Gal6 or ST6Gal1 to reinstall ST-specific sialylation capacities. Glycan schemes display representative examples of non-sialylated glycans expressed in HEK^ΔSia^N cells (tri-antennary N-glycans), HEK^ΔSia^O cells (core 1 and 2 O-glycans) or HEK^ΔSia^GSL cells (glycosphingolipid core structures of (*neo*)lacto-, ganglio-, and globo-series and Lac-Cer). Reported specificities of STs for either type I/II LN (Galβ1-3/4GlcNAc), type III LN (Galβ1-3GalNAc) or Lac-Cer (Galβ1-3Glc-Cer) are displayed. Not shown are ST3Gal3 and 6 which both act on type I/II LN. The cell-based array can be screened for virus infection (luciferase reporter assay) or virus binding (FACS analysis). In parallel, secreted glycoproteins (LAMP1 or Glycophorin A) carrying a biotinylation-tag (BAP-tag) can be transfected in the cell array to produce a specifically glycosylated protein library for binding analysis using biolayer interferometry (BLI). (B–D) Lectin binding to HEK^WT^ cells (WT) and glycoconjugate-specific cell lines (color-coded) transfected with specific STs as indicated on radar plots. Empty vector-transfected cells (no ST) are taken along for detection of Sia-independent background binding. According to the manufacturer Pan-Lec binds to any terminal located Sia and 2-3-Lec binds to α2-3-linked terminal Sia. SNA binds to α2-6-linked Sia on Galβ1-4GlcNAc (type II LN). (E–H) Lectin binding, determined by BLI, to LAMP I glycoprotein expressed in the glyconjugate-specific cell lines (color-coded) transfected by specific STs. MAL I binds to α2-3-linked terminal Sia on type II LN and MAL II binds to α2-3-linked terminal Sia on type III LN. ECA binds to terminal type II LN. (I–L) Lectin binding, determined by BLI, to GPa glycoprotein expressed as described in (E–H). Data are represented as mean.

Evolution toward utilization of N-glycan receptors as observed previous, coincides with evolution toward increased specificity for 2-6 Sia receptors.^13^ To dissect the contribution of Sia-linkage type and carrier glycoconjugate structures, an extended cell-based glycan array displaying specific sialoglycoconjugate assemblies was generated. HEK^ΔSia^ cells, deficient in all β-galactoside α2-6 and α2-3 STs,^27^ were modified (Figure 1A), to generate glycoconjugate-specific cell lines HEK^ΔSia^N, HEK^ΔSia^O, and HEK^ΔSia^GSL. From these, a sialoglycoconjugate cell-display library was generated by transient, one-by-one, reconstitution of sialylation capacities of individual STs (Figure 2A).

The cell library was characterized by lectin profiling of cell surface-displayed glycans by FACS (Figures 2B–2D, gating strategy in Figure S2). The ST/cell combination providing the highest binding of a specific lectin was used for normalization (e.g., ST3Gal3-transfected HEK^ΔSia^N cells for PAN-Lec, Figure 2B) of data plotted in radar diagrams. Transfected STs were plotted on the axes and data points are connected by a cell line-specific color (e.g., red for HEK^ΔSia^N cells). ST6Gal1 and ST3Gal3, 4, and 6 display substrate specificity for terminal N-acetyllactosamine (LN) which is here restricted to type II LN (Galβ1-4GlcNAc) as type I LN synthesis (Galβ1-3GlcNAc) does not occur in HEK^WT^ cells.^11^ Terminal LN is abundant on complex N-glycans but also on (*neo*) lacto-series glycolipids and core 2 O-glycans (B3GNT3 is not expressed,^11^ prohibiting core3/4 formation). Although Pan-specific Lectenz (Pan-Lec; binding terminal Sia) suggested strong activity of ST6Gal1 and ST3Gal3, 4 and 6 on N-glycans (HEK^ΔSia^N) but weaker activity on O-glycans (HEK^ΔSia^O) and GSLs (HEK^ΔSia^GSL) (Figure 2B), linkage-type specific lectins SNA (2-6Sia) and 2-3Sia-specific Lectenz (2-3Lec) detected, respectively, efficient ST6Gal1-induced sialylation of all three glycoconjugates classes (Figure 2D) and ST3Gal3-, 4-, and 6-induced sialylation of O-glycans (Figure 2C). Of note, ST activity may affect, or be affected by, fucosylation or sulphation of terminal LNs that in turn may influence IAV binding but this was not yet further investigated. ST3Gal1 activity, installing 2-3Sia on type III LN (Galβ1-3GalNAc) on O-glycans, was only detected by Pan-Lec (Figure 2A), implying 2-3 Lec binding to be restricted to sialylated N-glycans. GM3 synthesis by ST3Gal5 acting on lactosyl-ceramide was hardly detectable by lectin binding.

In summary, sialylation of type II LN by ST6Gal1 and ST3Gal3/4/6 occurs on all three types of glycoconjugates in HEK^WT^ cells. ST3Gal1 installed Sia on O-glycans and ST3Gal5 possibly installed minor levels of Sia on GSLs (e.g., as GM3, GM4) on HEK^ΔSia^GSL cells.

Secreted glycoprotein probes representative for N-linked glycoproteins (LAMP1; lysosomal associated membrane protein, 18 N-glycans) or typical O-linked mucin domains (GPa; glycophorin A, 17 O-glycans) were co-expressed with STs in the glycoconjugate-specific cell lines to produce a glycoprotein array parallel to the cell array for comparative binding studies. Lectin profiling by biolayer interferometry (BLI) was done using MAL I (type II Siaα2-3LN), MALII (type III Siaα2-3LN), and SNA (type II Siaα2-6LN) for quantifying specific sialylation and ECA (type II LN termini) for detection of a corresponding decrease in non-sialylated termini. In absence of co-transfected STs, ECA detected type II LN on N-glycans (Figure 2E, LAMP1) but hardly on O-glycans (Figure 2I, GPa). Complete reduction of ECA binding and increase of MAL I binding indicates complete capping of type II LN termini on LAMP1 by ST3Gal3 and 4 (Figures 2E and 2F). In contrast, ECA signal was only ∼60% reduced by ST6Gal1 (even at a 50-fold increased transfection dose, Figure S3), confirming that ST6Gal1 is more active on the α3 than the α6 branch of N-glycans.^31^ MAL II binding showed that the few O-glycans on LAMP1^32^ can be sialylated by ST3Gal1 and 2 in HEK^ΔSia^O cells (Figure 2G, green line). The abundant O-glycans on GPa were sialylated by ST3Gal1 and 2 in HEK^ΔSia^O cells (Figure 2K, green line) and even more efficiently in HEK^ΔSia^ cells (black lines). Albeit at low levels, binding of MAL I (Figure 2J) and SNA (Figure 2L) to GPa co-expressed with ST3Gal3, ST3Gal4, or ST6Gal1 in HEK^ΔSia^O cells indicated sialylation of type II LN termini on O-glycans.

In conclusion, lectin profiling of cells and cell-produced glycoprotein probes displays distinct profiles for the different cell and ST combinations that largely correspond to patterns expected on basis of their reported specificities.

### Sialylated type II LN on each glyconjugate group supports efficient infection

**Figure 3:**
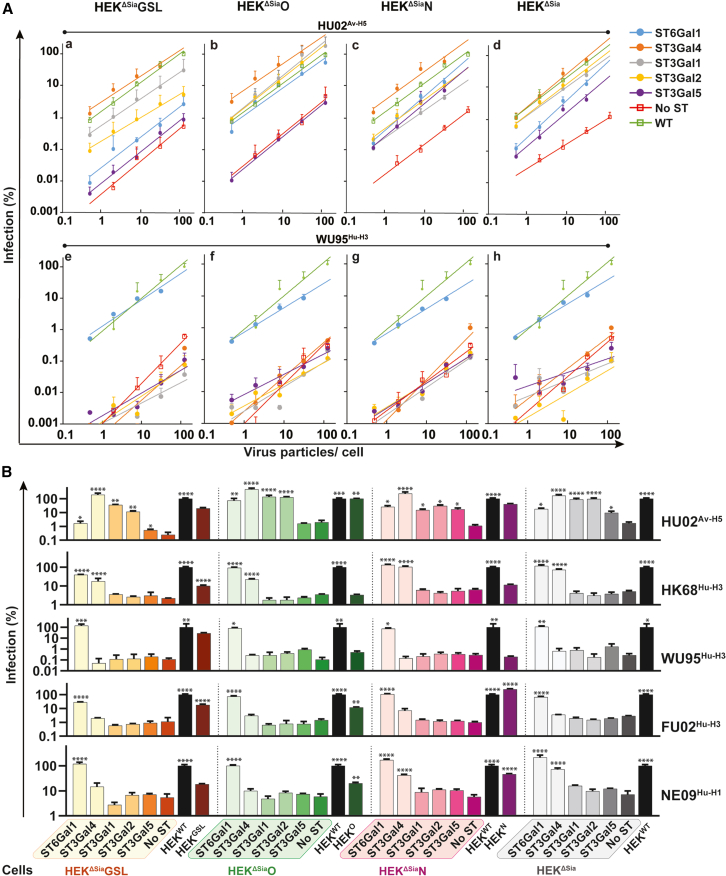
Determination of sialoglycoconjugate-specific infection efficiency of avian and human IAV strains (A) Infection efficiency of avian H5N1 (HU02^Av−H5^) and human H3N2 (WU95^Hu−H3^) in glycoconjugate-specific cell lines transfected with the indicated STs. Infection by serially diluted virus doses (x axis, virus particles/cell) was determined by induction of Gaussia luciferase activity. Values are normalized to infection levels obtained in HEK^WT^ cells at the highest infection dose (y axis) and fitted curves fitted (y = *a*x^*b*^) are displayed (statistics in Table S2). Plots of infection efficiency of human H1N1 (NE09^Hu−H1^) and H3N2 strains WU95^Hu−H3^ and FU02^Hu−H3^ are shown in Figure S5. (B) Infection efficiency at an infectious dose of 1 particle/cell (calculated from (A) and Figure S5; normalized to HEK^WT^) is displayed in bar diagrams. Significant enhancement by a specific ST over vector-transfected control (No ST) is marked (*p* < 0.0001, ∗∗∗∗*p* < 0.001, ∗∗∗*p* < 0.005, ∗∗*p* < 0.05, ∗.). Data for relative infection efficiency into HEK^GSL^, HEK^O^, HEK^N^ are taken from Figure 1. Data are represented as mean ± SEM.

Infection efficiency supported by specific sialo-glycoconjugate receptors was tested for avian H5N1 (HU02^Av−H5^), pandemic H1N1 (NE09^Hu−H1^), and three H3N2 strains (dual 2–3/2-6Sia-specific pandemic HK68^Hu−H3^, 2-6Sia-specific WU95, and slightly dual-specific FU02^13^). Sialyltransferases were expressed to levels supporting saturating levels of infection as previously shown^17^ or as determined here (Figures S4A–S4F). Comparison of infection into transiently transfected HEK^ΔSia^ cells with stable knock-ins of ST6Gal1 or ST3Gal4 in HEK^ΔSia^ cells validated the use of the cell-based glycan array as specificity and efficiency of infection by strains WU95^Hu−H3^ and HU02^Av−H5^ was similar in both cases (Figure S4G). Curves (fitted to power function y = *a*x^*b*^; statistics in Table S2) for 2-3Sia-specific HU02^Av−H5^ and 2-6Sia-specific WU95^Hu−H3^ are shown in Figure 3A and for all strains in Figure S5. To assist quantitative comparison of infection levels supported by the different glycan assemblies, we plotted infection levels at a dose of 1 particle/cell in bar diagrams (Figure 3B).

**Figure 4:**
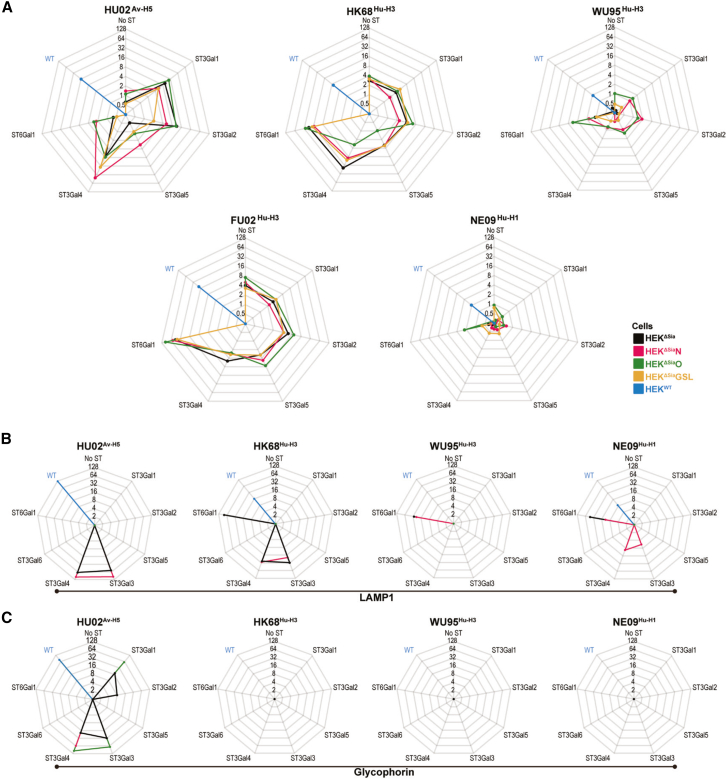
Virus binding to sialoglycoconjugates installed by transfection of specific STs on different glycoconjugates (A) Cell binding efficiency of virus strains (25 particles/cell) was determined by FACS and normalized to the highest observed value (FU02 binding to ST6Gal1-transfected HEK^ΔSia^O cells is 100%). Radar plots, with transfected STs indicated at the corners and datapoints (log2 scale) connected by color-coded lines for each specific cell line, display the relative binding efficiency. No ST displays background levels observed for empty vector-transfected cells and WT shows binding to HEK^WT^ cells. Virus strains that were tested are avian H5N1 (HU02^Av−H5^), human H1N1 (NE09^Hu−H1^), and human H3N2 strains HK68^Hu−H3^, WU95^Hu−H3^, and FU02^Hu−H3^ (B) Virus binding, determined by BLI, to LAMP1 glycoprotein expressed in the glyconjugate-specific cell lines (color-coded) transfected by specific STs. Values were normalized to HU02^Av−H5^ binding to LAMP1 isolated from ST3Gal4-transfected HEK^ΔSia^N cells (C) virus binding, determined by BLI, to GPa glycoprotein expressed as described for (B). Values were normalized to HU02^Av−H5^ binding to LAMP1 isolated from ST3Gal4-transfected HEK^ΔSia^O. Data are represented as mean ± SEM.

Sialylated N-glycans are considered as the prime IAV receptors. In accordance, infection levels similar or even higher as observed in HEK^WT^ or HEK^N^ cells were obtained in HEK^ΔSia^N cells transfected with ST3Gal4 or ST6Gal1 (Figure 4B). Variable support for IAV infection by O-glycans has been reported, likely reflecting the use of different strains and cell types, whereas support by GSLs has hardly been explored. Here, as for N-glycans, sialylation of type II LN on O-glycans or GSLs by ST3Gal4 efficiently supported infection of HU02^Av−H5^ and, somewhat less, HK68^Hu−H3^. On the other hand, sialylation of O-glycans or GSLs by ST6Gal1 supported infection of all human strains to 35% to 100% of wild-type levels. A selectivity index plot (ST3/ST6 infection efficiency at 1 particle/cell) shows (Figure S6) that relative infection levels supported by ST3Gal4 or ST6Gal1 matched previously determined^13^ relative affinities for 2-3 sialyllactosamine and 2-6 sialyllactosamine (K_D_ 2–6/K_D_2-3). Remarkably, infection of cells by WU95^Hu−H3^ was not increased upon transfection with ST3Gal4 even though WU95^Hu−H3^ and FU02^Hu−H3^ displayed similar 2-3Sia/2-6Sia K_D_ ratios and infection of FU02^Hu−H3^ was increased by ST3Gal4 transfection of all four cell lines.

In conclusion, ST3Gal4 and ST6Gal1 can efficiently sialylate the type II LN termini present on N-glycans, O-glycans and glycolipids as shown by induction of specific lectin binding to cells and glycoprotein probes (Figure 2). Sialylated type II LN on each of the three glycoconjugate types can efficiently support IAV infection in good correlation with receptor binding affinity. Thus, the gradual change in utilization of O-glycans and GSLs by H3N2 strains (Figure 1) may result from underrepresentation of 2-6Sia on these glycoconjugates in HEK^WT^ cells.

### Sialylation of O-glycans or GSLs by ST3Gal1 or 2 supports efficient HU02^Av−H5^ infection

ST3Gal1 and ST3Gal2 have been reported to sialylate type III LN with a preference for, respectively, O-glycans (core 1 and 2) or GSLs (gangliosides and globosides) (Figure 2A). Even though they induce only limited PAN-specific lectin binding when transfected into HEK^ΔSia^O cells (19% for ST3Gal1, 6% for ST3Gal2, Figure 2B), they induced efficient HU02^Av−H5^ infection of HEK^ΔSia^O cells (138% by ST3Gal1 and 129% by ST3Gal2, relative to HEK^WT^ cells) and HEK^ΔSia^GSL cells (36% and 12%) albeit less efficiently (Figures 3A-a, 3A-b, and 3B). Enhanced HU02^Av−H5^ infection of HEK^ΔSia^N cells upon transfection by ST3Gal1 (15% of WT) or ST3Gal2 (30% of WT) was unexpectedly observed (Figure 3A-c) which suggests that ST3Gal1 and 2 may indeed use type II LN substrates on N-glycans. Transfection of HEK^ΔSia^GSL cells with ST3Gal5, known as sialyllactosyl-ceramide (GM3) synthase (Figure 2A), results in ∼2.5-fold enhanced HU02^Av−H5^ infection but, intriguingly, infection was more efficiently enhanced by ST3Gal5 in HEK^ΔSia^ (∼6-fold) and HEK^ΔSia^N (∼15-fold) cells. Of note, ST3Gal5 also produces GM4 by sialylation of galactosyl-ceramide,^33^ which is present in all cell lines, and may act on additional substrates. In conclusion, sialoglycoconjugates installed by ST3Gal1 and ST3Gal2 on O-glycans or GSLs are major functional receptors for avian HU02^Av−H5^ but not for human IAVs. GM3 and GM4 likely support infection of HU02^Av−H5^.

### Correlation of virus binding to infection

Binding efficiency to sialoglycan repertoires installed by a specific ST was examined by virus binding to cell-based glycan arrays and derivative glycoprotein arrays. Virus binding to cells (Figure 4A) was quantified by FACS using a monoclonal antibody directed against a highly conserved HA-stem epitope (for gating strategy see Figure S7). Virus binding rate to glycoproteins LAMP1 (Figure 4B) or GPa (Figure 4C) was measured by BLI. For each assay, data were normalized to the highest signal (see legend Figure 4). In general, HU02^Av−H5^ displayed the strongest binding to ST3Gal-transfected cells whereas human IAV strains preferred binding to ST6Gal1-transfected cells (Figure 4A). Confirming a reported dual-specific binding affinity,^13^ HK68^Hu−H3^ did bind equally to ST3Gal4 and ST6Gal1-transfected cells. As reported for binding to synthetic glycans,^4^ NE09^Hu−H1^ displayed weak cell binding. H3N2 strains isolated after 2000 have been shown to bind most efficiently to 2-6Sia on biantennary synthetic N-glycans displaying branches extended by multiple LN repeats. This potentially enables bivalent interaction with a single HA trimer^16^ to compensate for the low monovalent binding affinity.^13^ However, strong binding of FU02^Hu−H3^ to ST6Gal1-transfected HEK^ΔSia^O cells (Figure 4A) suggests that densely packed O-glycans on mucin domains may also compensate for a low monovalent binding affinity. In concordance with strong FU02^Hu−H3^ binding to ST6Gal1-transfected HEK^ΔSia^GSL cells, HEK cells have been reported to display extended neolacto-type GSLs.^34^ Transfection of HEK^ΔSia^O cells or HEK^ΔSia^GSL cells with ST3Gal1 or 2 only induced enhanced binding of HU02^Av−H5^ but not of human strains.

**Figure 5:**
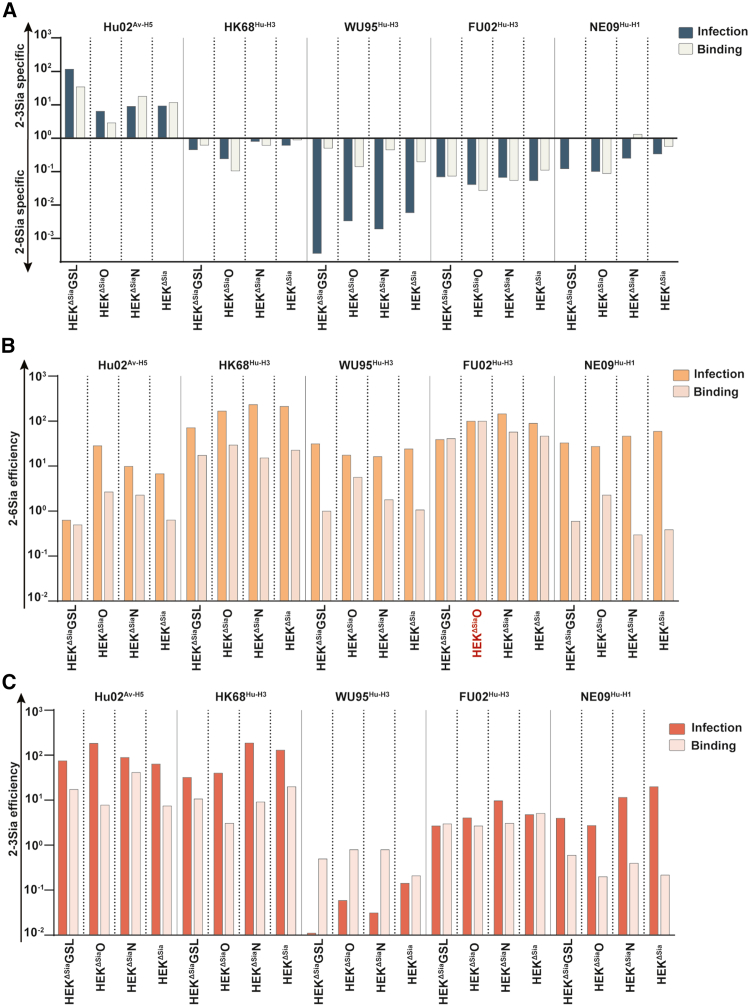
Comparison of binding and infection specificity and of infection efficiency relative to binding (A) Infection specificity (dark color) and binding specificity (light color) were defined as the values obtained for ST3Gal4-transfected cell lines divided by the values obtained for ST6Gal1-transfected cell lines (values taken from Table S3) for the three glycoconjugate cell lines and HEK^ΔSia^ cells. Virus strains are indicated above the bars. (B and C) Efficiency of infection (dark color) and binding (light color) of ST6Gal1 transfected cells (B) or ST3Gal4 transfected cells (C) was compared. The infection and binding of ST6Gal1-transfected HEK^ΔSia^O cells by FU02^Hu−H3^ was taken as a standard (100%) to which all values were normalized (indicated in red). Virus strains are indicated above the bars. Data are represented as mean.

For direct comparison, the virus and lectin binding data and infection data (Figures 2, 3, and 4) are assembled in Table S3a–S3d. Receptor binding specificity was compared to receptor-specific infection efficiency by plotting, for both, the 2-3Sia/2-6Sia ratio derived from ST3Gal4-and ST6Gal1-transfected HEK^ΔSia^N, HEK^ΔSia^O, HEK^ΔSia^GSL, and HEK^ΔSia^ cells. As shown previously, ST3Gal4 and ST6Gal1 install Sia on type II LN termini present on all three glycoconjugate classes. Infection of HEK^ΔSia^ cells displayed 2-3Sia-specificity for HU02^Av−H5^, dual-specificity for HK68^Hu−H3^, high 2-6Sia-specificity for WU95^Hu−H3^, and moderate 2-6Sia-specificity for FU02^Hu−H3^ and NE09^Hu−H1^ (Figure 5A, dark bars). The three glycoconjugate groups displayed the same specificity profile although considerable differences in absolute specificity were observed. For instance, HU02 infection supported by GSLs is ∼18-fold more specific as infection supported by O-glycans. Binding specificity (Figure 5A, light bars) is, with some variation, similar to infection specificity except for WU95 for which infection specificity is much larger than binding specificity (up to ∼1000-fold for GSLs).

A poor correlation between *in vitro* binding strength and infection efficiency of IAVs has frequently been reported.^8^^,^^14^^,^^35^^,^^36^^,^^37^ Here, we compared virus binding to ST3Gal4- or ST6Gal1-transfected cells with infection. FU02^Hu−H3^ was relatively efficient in both binding and infection for ST6Gal1-transfected HEK^ΔSia^O cells and therefore used for normalization of all strains in Figure 5B (2-6Sia) and Figure 5C (2-3Sia). Correlation between infection and binding appears to be strain-specific. For NE09^Hu−H1^, infection efficiency was relatively high in comparison to the low binding efficiency (e.g., more than 100-fold higher efficiency of infection over binding after normalization to FU02^Hu−H3^). For HU02^Av−H5^ and HK68^Hu−H3^ this difference was smaller. In contrast, WU95^Hu−H3^ infection of ST3Gal4-transfected cells (Figure 5C) was relatively low in comparison to binding but relatively efficient in ST6Gal1-transfected cells (Figure 5B).

Human IAV strains HK68^Hu−H3^, WU95^Hu−H3^, and NE09^Hu−H1^ were efficiently bound by 2–6 sialylated N-glycans on LAMP1 (Figure 4B) in accordance with their efficient binding to ST6Gal1-transfected cells (Figure 4A). FU02^Hu−H3^ did not show any binding in BLI assays but did efficiently bind to ST6Gal1-and ST3Gal4-transfected HEK^ΔSia^N cells (Figure 4A). These results, as well as the lack of binding of human IAVs to GPa (Figure 4C) clearly demonstrate the higher binding sensitivity of cell-based glycan arrays and, with some exceptions, a better correlation to infection of the same arrays. In conclusion, screening of IAV virus binding and infection with a glycoengineered cell-based array provides for highly sensitive analysis of receptor specificity displaying a good correlation between binding and infection. The use of specific glycoprotein BLI-based arrays is less sensitive in detecting binding, but has been shown to provide elaborate options for variation of binding conditions, receptor density, and comparative studies of the kinetics of virus binding and NA-dependent release.^38^

## Discussion

IAV infection involves consecutive stages of virus-receptor interaction including (1) initial cell surface binding, (2) rolling motility over the cell surface and (3) stalling at enigmatic sites that support endocytic entry. Infection of a HEK293 cell-based sialoglycan library enabled functional screening for receptor assemblies supporting these steps. Even at a physiologically relevant virus dose of one particle/cell, sub-assemblies of sialoglycans installed on N-glycans, O-glycans or GSLs efficiently supported infection, emphasizing the extreme sensitivity of the screen. Further, we discuss major findings, strengths, and weaknesses of the system.

### The role of N-glycans, O-glycans, and glycosphingolipids in IAV infection

Due to low affinity of HA-Sia interactions, IAV binding assays have mostly identified “strong binding receptors”, conveying the view that N-glycans are the major IAV receptors.^16^^,^^19^^,^^39^ Still, a consensus for support of IAV infection by complex assemblies of O-glycans and GSLs is lacking^18^^,^^19^^,^^20^^,^^21^^,^^22^ and “poor binding” viruses were shown to infect cells efficiently.^8^^,^^14^^,^^35^^,^^36^^,^^37^ Here, installing different Sias on distinct glycoconjugates uncovered a broad range of N-glycan, O-glycan, and GSL receptors supporting efficient infection by avian as well as pandemic and seasonal human IAV strains. Support of human IAV strain infection was restricted to N- and O-glycans and GSLs displaying α2-6Sia- and, in cases, also α2-3Sia-capped type II LN termini. Glycomic analysis of human respiratory epithelia has identified such sialylated termini on N- and O-glycans and (*neo*)lacto subclass GSLs.^8^ However, quantitative analysis of Sia-linkage type distribution is still required to assess their *in vivo* potential to contribute to infection of 2-6Sia-specific and dual-specific human IAVs. Avian H5N1 infection was supported by α2-3Sia-capped type II LN, but also by sialylated type III LN (Galβ1-3GalNAc) that is abundant in human mucus and may therefore act as decoy receptor limiting zoonotic transfer of avian IAVs.

### Evolution of receptor utilization by human seasonal H3N2 strains

Remarkably, in presence of the diversity of STs expressed by HEK^WT^ cells, O-glycan or GSL receptors displayed declining support for human seasonal H3N2 strains since 1968, which was inversed after 1995 (Figure 1). However, HEK^WT^ cells abundantly display 2-3Sias and 2-6Sias on N-glycans, whereas O-glycans and GSLs mostly display only 2-3Sias.^11^ Nevertheless, specific reconstitution of HEK^ΔSia^O or HEK^ΔSia^GSL cells with ST6Gal1 showed all H3N2 strains to be efficiently supported by 2-6Sia-capped O-glycans or GSLs (Figure 3). Thus, changes in support of infection of H3N2 strains by O-glycans and GSL rather reflects gradual evolution from dual- to 2-6-Sia linkage type preference as previously shown for N-glycans.^13^ Reversion to the avian-like HK68^Hu−H3^ pattern of receptor utilization after 1995 makes this unlikely to be an adaptation to the human receptor repertoire. Antigenic change by amino acid substitutions near the receptor binding site^40^ more likely drives altered receptor utilization. Ambiguous receptor utilization by IAV likely expands options for antigenic variation.

### Correlation of virus binding strength and specificity to infection efficiency

The positive correlation between binding and infection in relation to Sia linkage type specificity (Figure 4A), was less obvious when comparing absolute binding efficiency and infection efficiency (Figures 4B and 4C) and corroborates previous observations.^8^^,^^14^^,^^35^^,^^36^^,^^37^ Several receptors displayed poor binding avidity but efficiently supported infection. Possibly, the consecutive stages of virus-receptor interaction involved in infection are differentially affected by affinity for the heterogeneous receptor repertoire encountered at the cell surface. For example, initial cell surface binding may be promoted by high affinity HA-Sia interactions^13^ whereas receptor-dependent rolling over the cell surface to entry-competent sites^41^ may be slowed down by tight interactions. Also, receptor diversity contributes to a heteromultivalent binding mode^17^ by which low-affinity receptors reduce the minimal density of stronger-binding receptors required for establishing the initial monovalent interaction. Such a binding mode may further contribute to different receptor requirements for efficient binding, rolling, and entry.

### The role of NA and the HA/NA balance

NA activity is crucial to IAV infection in being a driver of virus motility that is essential to become located at entry-competent sites.^38^^,^^41^^,^^42^ Motility requires balanced HA binding and NA activity. Whereas there is ample proof for the existence of such a balance,^43^ the contribution of NA to this balance remains largely undetermined. Likely, different NAs display different activity spectra on assemblies of synthetic glycans or cell surface-displayed glycan repertoires but assays to analyze this independently of HA are lacking. Expectedly, specificity changes in NA are among the underlaying factors explaining the, occasional, poor correlation between binding strength and infection efficiency. Improved understanding of the HA/NA balance in relation to a specific cell surfaced-displayed receptor repertoire is therefore required.

### Implications of receptor diversity and accessibility for entry pathway selection

IAV enters cells via endocytic mechanisms, including clathrin-mediated endocytosis,^44^ macropinocytosis,^26^ and likely other pathways.^42^ Specific protein receptors, presumably bound by IAV via their attached sialoglycans,^29^ have been implied in initiating endocytic pathways upon activation of diverse signaling cascades.^29^^,^^44^ A major implication of the current study is that, even though these events likely display glycoconjugate class-specific features, each of the three glycoconjugate classes can individually support highly efficient entry. Efficient infection of cells displaying only sialylated GSLs dismisses the absolute need for Sia-dependent engagement of proteinaceous signaling receptors. This corresponds with previous observations of redundant endocytic pathways^26^^,^^42^ and suggests a role for other signaling cues originating, for instance, from virus binding-induced membrane curvature that can be sensed by specific intracellular signal transduction activators.^43^^,^^45^

The structural basis of receptor specificity^1^^,^^2^ does not clarify the dynamic interactions leading to cell entry on a heterogeneous receptor surface. Initial binding and virus motility preceding entry is crucially dependent on the affinity of diverse short-lived HA-Sia interactions (t_1/2_ = 0.8s)^13^ as well as receptor density and accessibility to sustain dynamic heteromultivalent interactions. Obviously, in comparison to HEK^WT^ cells, the cells of our library may display glycans at altered density and accessibility to virus particles. N-glycans and especially GSLs of the (*neo*)lacto subclass were shown to display Sia on glycan branches of extended length at human respiratory epithelia,^8^^,^^11^^,^^46^ and may likely be as accessible to viruses on glycan-crowded wild-type cells as on our library cells. Virus accessibility to specific glycans present at epithelial surfaces has hardly been experimentally addressed. Notably, even short glycans such as ganglioside GD3 have been shown to function as efficient receptors for coronaviruses, such as OC43 and HKU1 when expressed on wild-type HEK293T cells^47^ and it cannot *a priori* be concluded that short glycans are inaccessible to virus binding *in vivo*.

In conclusion, genetic dissection of specific glycan assemblies on cells enables highly sensitive screening for identification of functional IAV receptors. Further knock-out/knock-in of glycosyltransferases can refine structural requirements and the cells can be used for detailed analysis of the receptor requirements for induction of specific endocytic virus entry pathways. Sialoglycans not supporting entry may still participate in binding via heteromultivalent binding^17^ and can be identified by co-transfection of different STs. Libraries produced by transient transfection are ideally suited for such a combinatorial approach. The current results support the point of view that utilization of a wide range of receptors will expand the capacity of gradual evolution of receptor preference in response to host switching and antigenic change.

### Limitations of the study

Our study builds on cell libraries^11^ used to characterize Sia binding specificities of Siglecs.^27^ While presenting sialoglycans on glycoconjugates in the natural context of the surface of living cells, there are limitations. Sialoglycoconjugate cell-display libraries crucially provide restricted glycan repertoires installed by a specific ST on single glycoconjugate classes. The HEK293 glycoengineered arrays do not yet comprehensively display all variations of the respiratory tract glycome (e.g., type I LN not expressed). Also, displayed patterns of N- and O-glycans are limited by the repertoire of proteins expressed. The same though, applies to classical IAV binding studies using re-sialylation of erythrocytes.^48^^,^^49^ Cell surface glycomes result from balanced activities of multiple glycosyltransferases working in concert or in competition for available acceptor substrates. Exogenous ST expression may affect endogenous glycosylation efficiencies, e.g., overexpression of ST6GAL1 was found to reduce poly-LacNAc extension of N-glycans,^50^ which is known to reduce binding of human H3N2 strains isolated after 2000.^16^ However, specific tuning of glycosyltransferase expression levels will extend the use of the cell-display platform to the analysis of such modifications. E.g., we previously showed that elongation of glycan chains with additional LN repeats by overexpression of specific galactosyl- and glucosaminyl-transferases restored efficient FU02^Hu−H3^ binding.^13^ This strategy can also be used to determine effects on IAV binding and infection by features such as linkage-types of glycosidic bonds, branching, chain length, and ST8-dependent poly-sialylation. Obviously, the platform can be applied to a wide range of glycan-binding viruses.

Another limitation of cell line-based systems in comparison to infection at epithelia is the absence of a protective mucus layer. O-glycans are abundant on secreted mucins, which may function as protective decoy receptors.^24^^,^^25^^,^^51^^,^^52^ Human secreted mucins typically carry sialylated O-glycans with varying core branching structures (cores 1–4) dependent on cell types and tissues. O-glycans can display LacNAc termini that may be capped by 2–3 or 2-6Sia linkages. Here, we clearly provide evidence that core 2 O-glycans can be capped by 2-6Sia mediated by transient expression of ST6GAL1 (Figure 2D). Whereas mucins are generally considered to be rich in 2-3Sia, Sia-linkage type-specific glycomic analyses of mucins and mucus are generally lacking and such studies are crucial to understanding the barrier function of mucus for IAV infections. Clearly, there is no strict requirement for human IAVs to prevent the observed interaction with 2-3Sia-rich O-glycans (HK68^Hu−H3^ and late H3N2 strains) and presence of 2-6Sia-capped O-glycans may provide additional decoy receptors.

Finally, a detailed analysis of the glycome of all the glycoengineered cells may refine some conclusions and provide additional insights. Currently, such techniques are not high-throughput and are therefore extremely resource demanding. Also, knowledge on the glycome will still remain largely inconclusive in exactly defining binding epitopes without further experimentation. Instead, this study relied on lectin profiling (Figure 2), general existing knowledge of human cellular glycosylation pathways^53^ and kinetic properties of human STs,^54^ and our previous analyses of outcomes of wide glycoengineering in HEK cells.^11^^,^^27^^,^^55^^,^^56^ In absence of detailed structural analyses of the diversity of 2–3/2–6 linked Sias, and yet very limited structural analyses of glycolipids, our use of HEK^ΔSia^ cells has clearly demonstrated that 2-3Sias and 2-6Sias can be efficiently exposed on the three main types of glycoconjugates. Quantification of relative abundancies of individual sialoglycans is difficult to perform but can, as performed here, be estimated from semi-quantitative lectin profiling. Future studies are needed to develop stable engineered cells lines with these sialoglycan variants allowing more detailed quantitative studies. For now, the results support the general conclusions drawn about the glycoconjugate and Sia linkage involved in supporting binding and infection of specific IAV strains.

## Resource availability

### Lead contact

Further information and requests for resources and reagents should be directed to and will be fulfilled by the lead contact, Dr. Erik de Vries (e.devries@uu.nl).

### Materials availability

Plasmids used in this study have been indicated in the key resources table. All unique cells generated in this study will be made available through the lead contact.

### Data and code availability

• Data: All data generated on which the figures are based are included in the manuscript or the supplemental information.
• Code: This study did not generate new codes.
• Other: any additional information required to reanalyze the data reported in this paper is available from the lead contact upon request.

## Acknowledgments

Support by a personal grant from the Chinese Scholarship Council to M.L. (201908350116) and X.W. (202006010036) was obtained. Support by the 10.13039/501100003554Lundbeck Foundation, 10.13039/501100009708Novo Nordisk Foundation (grants NNF24OC0088218 and NNF21OC0071658) and the 10.13039/501100001732Danish National Research Foundation (DNRF107) was obtained (Y.N. and H.C.). We thank Dr. Ron Fouchier (Viroscience, Erasmus Medical Center, Rotterdam, the Netherlands) for providing virus strains.

## Author contributions

Conceptualization: M.L., C.A.M.d.H., and E.d.V.; formal analysis: M.L. and E.d.V.; methodology: M.L., Y.N., H.C., C.A.M.d.H., and E.V.; investigation: M.L., X.W., M.D.B.G., and Y.N.; visualization: M.L., X.W., and E.d.V.; supervision: F.J.M.v.K., C.A.M.d.H., and E.d.V.; funding acquisition: F.J.M.v.K.; writing – original draft: M.L. and E.d.V.; writing – review and editing: M.L., Y.N., F.J.M.v.K., H.C., C.A.M.d.H., and E.d.V.

## Declaration of interests

Y.N. and H.C. have a financial interest in GlycoDisplay Aps, Y.N.’s and H.C.’s interests are reviewed and managed by the University of Copenhagen in accordance with their conflict of interest policies.

## STAR★Methods

### Key resources table

**Table:** 

REAGENT or RESOURCE	SOURCE	IDENTIFIER
**Antibodies**		

Alexa Fluor 488 goat-*anti*-human IgG	ThermoFisher	A-11013
Anti-HA antibody MEDI produced in HEK293F cells	This paper	N/A

**Bacterial and virus strains**		

A/Bilthoven/16190/68 (H3N2; HK68^Hu−H3^)	Koel et al.^40^	N/A
A/Bilthoven/21793/72 (H3N2; EN72 ^Hu−H3^)	Koel et al.^40^	N/A
A/Bilthoven/1761/76 (H3N2; VI75 ^Hu−H3^)	Koel et al.^40^	N/A
A/Bilthoven/2271/76 (H3N2; TX77 ^Hu−H3^)	Koel et al.^40^	N/A
A/Netherlands/233/82-193N (H3N2; BK79 ^Hu−H3^)	Koel et al.^40^	N/A
A/Netherlands/620/89 (H3N2; SI87 ^Hu−H3^)	Koel et al.^40^	N/A
A/Netherlands/179/93 (H3N2; BE92 ^Hu−H3^)	Koel et al.^40^	N/A
A/Netherlands/178/95-145K (H3N2; WU95 ^Hu−H3^)	Koel et al.^40^	N/A
A/Netherlands/427/98 (H3N2; SY97 ^Hu−H3^)	Koel et al.^40^	N/A
A/Netherlands/213/03 (H3N2; FU02 ^Hu−H3^)	Koel et al.^40^	N/A
A/Perth/16/2009 (H3N2; PE09 ^Hu−H3^)	Koel et al.^40^	N/A
A/duck/Hunan/795/2002(H5N1; HU02 ^Av−H5^)	Liu et al.^17^	N/A
A/Netherlands/602/2009 (H1N1; NE09^Hu−H1^)	Maines et al.^57^	N/A
pRluc-53CB3/T7	van der Schaar et al.^58^	N/A

**Chemicals, peptides, and recombinant proteins**		

Lipofectamine 2000 transfection reagent	Invitrogen^TM^	11668019
Lipofectamine 3000 transfection reagent	Invitrogen^TM^	L3000150
FuGENE 6 transfection reagent	Promega	E2691
Maackia Amurensis Lectin I (MAL I)	Vector Laboratories	B-1315-2
Maackia Amurensis Lectin II (MAL II)	Vector Laboratories	B-1265-1
Erythrina Cristagalli Lectin (ECA)	Vector Laboratories	B-1145-5
Biotinylated Sambucus Nigra Lectin (SNA)	Vector Laboratories	B-1305-2
Sambucus Nigra Lectin (SNA)	Vector Laboratories	L-1300-5
Biotinylated Pan-specific Lectenz	LectenzBio	SK0502B
Biotinylated α2,3-specific Lectenz	LectenzBio	SK2301B
Dulbecco’s PBS + Ca^2+^/Mg^2+^	Capricorn scientific	PBS-2A
DMEM High Glucose with L-Glutamine	Capricorn scientific	CA DMEM-HA
Opti-MEM + GlutaMAX	GIBCO	51985–026
Penicillin-Streptomycin	Thermo Fisher Scientific	15140122
Biotinylated LAMP1 (lysosomal membrane protein 1)	Liu et al.^17^	N/A
Biotinylated GPa (glycophorin A)	Liu et al.^13^	N/A
Fetal Bovine Serum	Biowest	S1520
Alexa Fluor647-conjugated streptavidin	Invitrogen	S21374
GFP-tagged CAS9PKBS	Addgene	Plasmid #68371
Oseltamivir Carboxylate	Hoffman-La Roche	Contact Roche
SA sensors biolayer interferometry	Sartorius	18–5020

**Critical commercial assays**		

Renilla luciferase assay system	Promega	E2810

**Experimental models: Cell lines**		

MDCK-II	ATCC	ATCC CRL-2963
HEK293 (HEK^WT^)	Narimatsu et al.^11^	N/A
HEK^N^ (ΔCOSMC/B4GALT5/6)	Narimatsu et al.^11^	N/A
HEK^O^ (ΔMGAT1/B4GALT5/6)	Narimatsu et al.^11^	N/A
HEK^GSL^ (ΔMGAT1/COSMC)	Narimatsu et al.^11^	N/A
HEK^ΔSia^ (ΔST6GAL1/ST6GAL2/ST3GAL1/ST3GAL2/ST3GAL3/ST3GAL3/ST3GAL5/ST3GAL6)	Bull et al.^27^	N/A
HEK^ΔSia^+ST6GAL1-KI (ΔST6GAL1/ST6GAL2/ST3GAL1/ST3GAL2/ST3GAL3/ST3GAL3/ST3GAL5/ST3GAL6+ST6GAL1)	Narimatsu et al.^11^	N/A
HEK^ΔSia^+ST3GAL4-KI (ΔST6GAL1/ST6GAL2/ST3GAL1/ST3GAL2/ST3GAL3/ST3GAL3/ST3GAL5/ST3GAL6+ST3GAL4)	Narimatsu et al.^11^	N/A
HEK^ΔSia^N	This paper	N/A
HEK^ΔSia^O	This paper	N/A
HEK^ΔSia^GSL	This paper	N/A

**Oligonucleotides**		

MGAT1 CRISPR/Cas9 gRNA GT13	Narimatsu et al.^59^	N/A
COSMC (C1GALTC1) CRISPR/Cas9 gRNA GT31	Narimatsu et al.^59^	N/A
B4GALT5 CRISPR/Cas9 gRNA GT7	Narimatsu et al.^59^	N/A
B4GALT6 CRISPR/Cas9 gRNA GT7	Narimatsu et al.^59^	N/A

**Recombinant DNA**		

pCDNA-ST3GAL1	Genscript, Piscataway	NM_0033033.3
pCDNA-ST3GAL2	Genscript, Piscataway	NM_006927.4
pCDNA-ST3GAL3	Genscript, Piscataway	NM_174963.3
pCDNA-ST3GAL4	Genscript, Piscataway	NM_006278.3
pCDNA-ST3GAL5	Genscript, Piscataway	NM_003896.4
pCDNA-ST3GAL6	Genscript, Piscataway	NM_006100.3
pCDNA-ST6GAL1	Genscript, Piscataway	NM_173216.2
pCDNA-ST6GALNAC2	Genscript, Piscataway	NM_006456
pCD5 BirA biotin ligase	Liu et al.^17^	N/A

**Software and algorithms**		

Excel	Microsoft	https://www.microsoft.com/en-ca/microsoft-365/excel
Octet Analysis studio 9.0.0.26	Sartorius	Octet® Software Download | Sartorius
GraphPad Prism 10.4.1	Graphpad	https://www.graphpad.com/
FlowJo v10	Beckman	https://www.flowjo.com
GloMax Discover System GM3000	Promega	https://www.promega.com
SH800S Cell Sorter	Sony Biotechnology	https://www.sonybiotechnology.com/instruments/sh800s-cell-sorter
NanoSight NS3000	Malvern Panalytical	https://www.malvernpanalytical.com

### Method details

#### Virus strains

The human H3N2 recombinant influenza strains used in this paper represent the antigenic clusters identified since 1968 and were generated and described previously.^40^ They are 2:6 reassortants containing the 6 internal gene segments of strain A/Puerto Rico/8/1934(H1N1) and the HA and NA gene segments of: A/Bilthoven/16190/68 (HK68^Hu−H3^), A/Bilthoven/21793/72 (EN72^Hu−H3^), A/Bilthoven/1761/76 (VI75^Hu−H3^), A/Bilthoven/2271/76 (TX77^Hu−H3^), A/Netherlands/233/82-193N (BK79^Hu−H3^), A/Netherlands/620/89 (SI87^Hu−H3^); A/Netherlands/179/93 (BE92^Hu−H3^); A/Netherlands/178/95-145K (WU95^Hu−H3^); A/Netherlands/427/98 (SY97 Hu-H3); A/Netherlands/213/03 (FU02 Hu-H3) and A/Perth/16/2009 (PE09 Hu-H3). A/Netherlands/602/2009 (NE09^Hu−H1^) is a 2009 field isolate of the pandemic H1N1 strain.^57^ A/duck/Hunan/795/2002 (HU02^Av−H5^) is a previously described avian strain.^17^ Virus strains were propagated by inoculating seed stocks into Madin-Darby canine kidney (MDCK)-II as described^55^ and stored at −80°C. Enterovirus strain RlucCVB3 was obtained by transfection of infectious clones pRluc-53CB3/T7^58^ and used at MOI 0.1 in infection and luciferase reporter assays.^17^

#### Nanoparticle tracking analysis (NTA)

Virus particle numbers were quantified by Nanoparticle Tracking Analysis (NTA) (NanoSight NS3000, Malvern Panalytical).^60^ Briefly, the virus solution was diluted in PBS to a suitable concentration for NTA analysis. The NanoSight NS300 captured five 60-s sample videos per analysis. These videos were then processed using the Nanoparticle Tracking Analysis 3.0 software to obtain quantitative data on both the number and size of virus particles.

Particle numbers were determined as the average of five measurements.

#### Cell culture

HEK^WT^ and HEK knock-out cell lines were maintained in DMEM (SIGMA) media supplemented with 10% fetal bovine serum (SIGMA) at 37°C and 5% CO2. HEK^ΔSia^ (ΔST3GAL1/2/3/4/5/6 and ST6GAL1/2) lacks all Siaα2-3Gal and Siaα2-6Gal glycotopes; HEK^N^ (ΔB4GALT5/6 and C1GALT1) only expresses N-linked glycans; HEK^O^ (ΔB4GALT5/6 and MGAT1) only expresses O-linked glycans; HEK^GSL^ (ΔC1GALT1 and MGAT1) only expresses glycosphingolipids.^11^^,^^55^

#### CRISPR/Cas9-targeted KO in HEK293 cells

CRISPR/Cas9 knockout cells were generated and authenticated as described.^11^ Briefly, CRISPR/Cas9 targeted KO in HEK^ΔSia^ cells was performed using the GlycoCRISPR resource containing validated gRNAs libraries for targeting of all human glycosyltransferases.^59^ HEK^ΔSia^ cells were grown in 6-well plates for 24h, and 1 μg of gRNA and 1 μg of GFP-tagged Cas9-PBKS were co-transfected using lipofectamine 3000 (Thermo-Fisher Scientific) following the manufacturer’s protocol. Transfected cells were harvested after 24h, and bulk-sorted based on GFP expression by FACS (SONY SH800). After one week of culturing, the bulk-sorted cells were pooled to further single-cell sorting into 96-well plates. The sorted clones were screened by Indel Detection by Amplicon Analysis (IDAA) and verified by Sanger sequencing.^61^

#### Single-round virus infection assays of HEK^WT^ cell lines

HEK^WT^-derived knock-out cell lines were performed using a luciferase reporter system employing the Gaussia luciferase-encoding vector pHH-Gluc as described.^26^ Briefly, cells were seeded in a 96-well plate (25,000 cells/well) to reach 90% confluency the following day at which cells were transfected with 25 ng pHH-Gluc in combination with different amounts of sialyltransferases (ST3Gal1-6, ST6Gal1 and ST6GalNAc2) as indicated in legends. Lipofectamine 2000 (Invitrogen) was used according to the manufacturer’s protocol. The cell culture medium was replaced with Opti-mem (Gibco) 6 h post-transfection. After 24 h, the transfected cells were infected with a 2-fold dilution series of virus particles as indicated in Figures 1 and 3. Infection was performed in absence of trypsin to be limited to a single round. Each condition was tested in triplicate, and two biological replicates were performed. At 17 h.p.i. samples from the supernatant were assayed for luciferase activity as described.^26^ For normalization, infection of Sia-independent enterovirus strain RlucCVB3, which was shown to give identical infection of the different cell types,^17^ was taken along in triplicate for every plate. An MOI of 0.1 for RlucCVB3 was used.

#### Expression and purification of biotinylated glycoprotein receptors

Recombinant lysosomal membrane protein 1 (LAMP1) and Glycophorin A (GPa) were expressed in different HEK^ΔSia^ Knockout cells. The transmembrane domain of LAMP1 and GPa was replaced by a BAP-tag, yielding a secreted and C-terminally biotinylated N-glycoprotein^17^ or O-glycosylation site, respectively. To install specific sialylation patterns on different HEK^ΔSia^ derived cells and the secreted glycoprotein LAMP1 or GPa, expression vectors encoding sialyltransferases (pCDNA-ST3Gal1-6 or pCDNA-ST6Gal1) (Genscript, Piscataway, NJ, USA, accession No: NM_0033033.3; NM_006927.4; NM_174963.3; NM_006278.3; NM_003896.4; NM_006100.3; NM_173216.2) and an expression vector (pCD5) encoding a BirA biotin ligase were co-transfected with an expression vector of LAMP1 or GPa. Transfection procedures, and recombinant protein purification by Ni-NTA binding chromatography have been described.^62^

#### Real-time virus-binding and lectin-binding assays by BLI

Real-time virus binding was studied by BLI analysis using an Octet RED384 (Fortebio). All experiments were carried out at 30°C in Dulbecco’s PBS with calcium and magnesium (PBS^+/+^) (Lonza) as standard assay buffer. BLI protocols have been described in detail.^62^ In short, SA biosensors were loaded with biotinylated glycoprotein LAMP1 or GPa with sialic aicds that were reinstalled by different STs as indicated in the legend. Glycoproteins were loaded to saturating levels and real-time virus association was examined for 900 s in the presence of 10 μM oseltamivir carboxylate (OC; Roche) to block NA activity. Absolute initial virus-binding rates were calculated and plotted (nm/10^9^ virus particles). LAMP1 and GPa sialylation levels were analyzed by lectin binding assays (Maackia amurensis lectin I (MAL I, Vector Labs) binds to α2-3Sia-Galβ1-4GlcNAc; Maackia amurensis lectin II (MAL II, Vector Labs) binds to α2-3Sia-Galβ1-3GalNAc; Sambucus nigra lectin (SNA, Vector Labs) binds preferentially to α2- 6Sia-Galβ1-4GlcNAc; and Erythrina cristagalli lectin (ECA, Vector Labs) binds to terminal LacNAc (Galβ1-4GlcNAc)). Specificities of lectins as mentioned in the text have been determined by glycan array analysis.^63^

#### Cell-binding assays

For lectin staining, HEK^WT^-derived KO cells were seeded in 12-well plates (1.5 × 10^5^ cells/well) and co-transfected with vector encoding specific sialyltransferases (pcDN3.1-SiaTs) or empty (pcDN3.1) using FuGene 6 (Promega, E2691) according to manufacturer’s protocol. Transfected cells were released by using Cell Dissociation Buffer (Gibco) at 48h post-transfection, followed by fixation with 3.7% paraformaldehyde (PFA) in PBS. Cells were blocked with high-purity BSA (Sigma, A7638) for 1h, and incubated for 1h on ice with Pan-lectenz (20 μg/mL) and Alpha 2,3-Specific Lectenz (20 μg/mL) (Lectenz Bio) that were pre-complexed with Alexa Fluor 647-conjugated streptavidin (1:1000) (Invitrogen by ThermoFisher Scientific) for 30 min, and with SNA-FITC (20 μg/mL) (Vector labs). All cells were resuspended in PBS for flow cytometry analysis (BC Cytoflex LX (Beckman). Mean fluorescent intensity (MFI) of the binding of sialic acids to positive and negative populations was quantified using FlowJo software (FlowJo LLC). The gating methods and representative flow cytometry gating strategies for lectin staining cells are shown in Figure S2.

For virus binding, cells were seeded in a T75 flask (3.0 × 10^6^ cells/flask) and the next day transfected with 8 μg of indicated plasmids (STs or empty pcDN3.1 vector) using FuGene 6 (Promega, E2691). Transfected cells were released by using 2.5% Trypsin-EDTA (Gibco) after washing at 48h post-transfection. Staining with viral HA to sialic acids was performed by incubating 2.0 × 10^5^ release cells with 10ul alive viruses (on average 5.6x10^8^ particles/ml) on ice for 30min, diluted with PBS in presence of 10 μM Oseltamivir Carboxylate (OC; Roche), followed by fixation with 3.7% PFA and blocking with 2% goat serum for 1h on ice. Fixed cells were complexed with human anti-HA antibody MEDI^64^ that was produced in HEK293F cells as described.^65^ Complexation was performed on ice for 1h and subsequent staining by Alexa Fluor 488-labeled goat anti-human IgG (ThermoFisher) for 1h. All cells were resuspended and washed in PBS in the presence of 10 μM OC for flow cytometry.

### Quantification and statistical analysis

All infection experiments were performed in triplicate with 2 biological replicates. Curve fitting of the virus entry data by a power regression model to the function y = ax^b^ was performed by the data analysis package of Microsoft Excel for Microsoft 365 MSO (Version 2302 Build 16.0.16130.20848) 32-bit. Data were first natural log transformed enabling linear log-log regression (ln y = b ln x + ln a) yielding R square, significance (p) and confidence intervals for a and b as listed in Tables S1 and S2. Two or three biological and technical replicates of BLI binding assays were performed. Statistical significance for bar diagram plots of Figure 3B was derived from the fitted curves of Figure 3A by using a modified Chi-squared method for determining the statistical significance of the difference between curves as described.^66^

## Supplemental information

Document S1. Figures S1–S7 and Table S1 and S2

Table S3. Normalized values of Lectin and Virus binding to Cells and Protein glycoprobes in comparison to normalized values of virus infection
