# Supplementary material for: Cell-based sialoglycan arrays for directly comparing influenza A virus receptor requirements for binding and infection
Source: iScience. 2025 May 3;28(6):112549. doi: 10.1016/j.isci.2025.112549 (PMC12145812; doi:10.1016/j.isci.2025.112549)
Supplement: Document S1. Figures S1–S7 and Table S1 and S2 [file mmc1.pdf]

iScience, Volume *28*

Supplemental information

Cell-based sialoglycan arrays for directly comparing influenza A virus receptor requirements for binding and infection

Mengying Liu, Xuesheng Wu, Martijn D.B. van de Garde, Yoshiki Narimatsu, Frank J.M. van Kuppeveld, Henrik Clausen, Cornelis A.M. de Haan, and Erik de Vries

Supplemental Information

From binding to infection: Receptor Requirements for Influenza A virus infection delineated using Cell-Based Sialoglycan Arrays

Mengying Liu, Xuesheng Wu, Martijn D.B. van de Garde, Yoshiki Narimatsu, Frank J.M. van Kuppeveld, Henrik Clausen, Cornelis A.M. de Haan, Erik de Vries

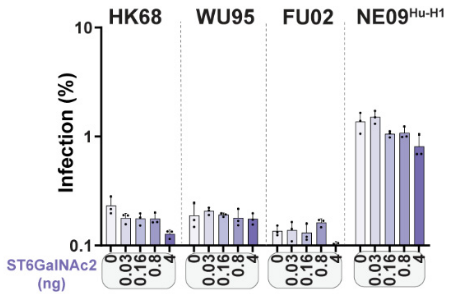

Figure S1 IAV infection in $HEK^{\Delta Sia}$ with ST6GalNAc2 overexpression. Related to Figure 1. Infection efficiency of human H3N2 and H1N1 in $HEK^{\Delta Sia}$ transfected with ST6GalNAc2. Amounts of transfected ST added are indicated (per well in 96-well plates. Infection efficiency (Gaussia luciferase activity) at an infectious dose of 100 particles/cell was determined. Values are normalized to infection of Sia-independent enterovirus strain RlucCVB3.

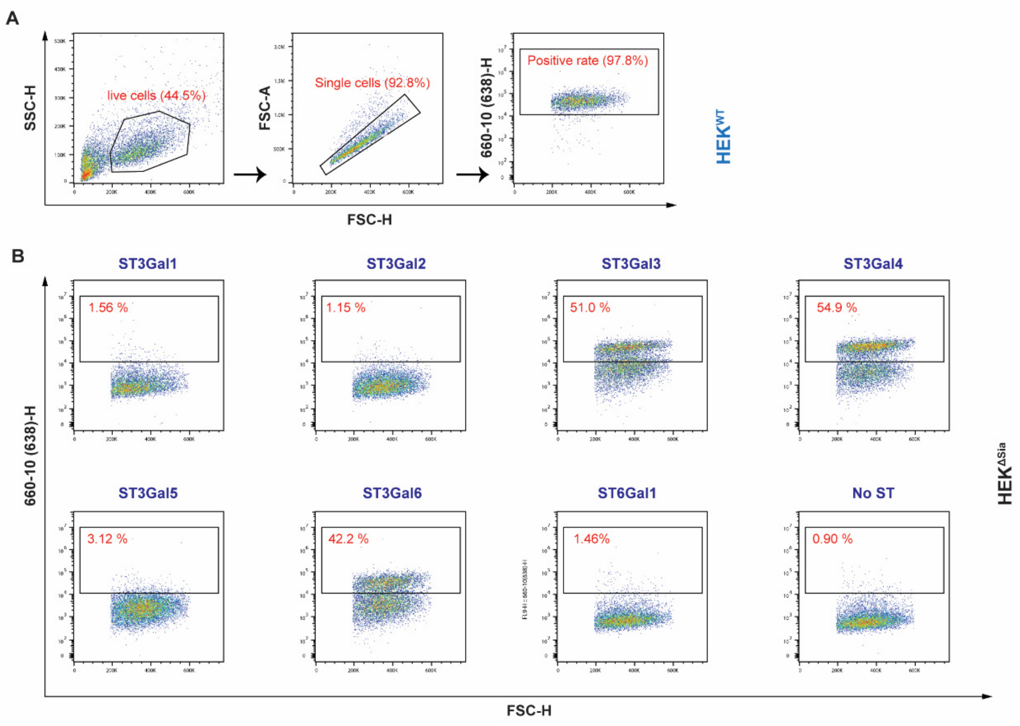

C

	HEK$^{\Delta Sia}$									HEK^{WT}
	ST3Gal1	ST3Gal2	ST3Gal3	ST3Gal4	ST3Gal5	ST3Gal6	ST6Gal1	ST6Gal2	No ST	
Mean (660-10(638)-H)	2904	2727	25842	31285	3918	18422	1839	1342	1786	52030
Positive rate (%)	1.56	1.15	51	54.9	3.12	42.2	1.46	0.9	1.68	97.8
Total fluorescence	4530.24	3136.05	1317942	1717546.5	12224.16	777408.4	2684.94	1207.8	3000.48	5088534

Figure S2 Gating strategy of 2-3Lec binding to sialoglycotopes. Related to Figure 2. (*A*) Representative flow cytometry gating demonstration of 2-3 Lectenz binding to HEK^{WT} cells showed nearly all cells to be positive. (*B*) HEK$^{\Delta Sia}$ cells transfected with the indicated sialyltransferases. Positive rate of 2-3 lectenz staining is indicated in the quadrant in red. (*C*) Total fluorescence in the red quadrant was determined and used for the radar plots in Figure 2.

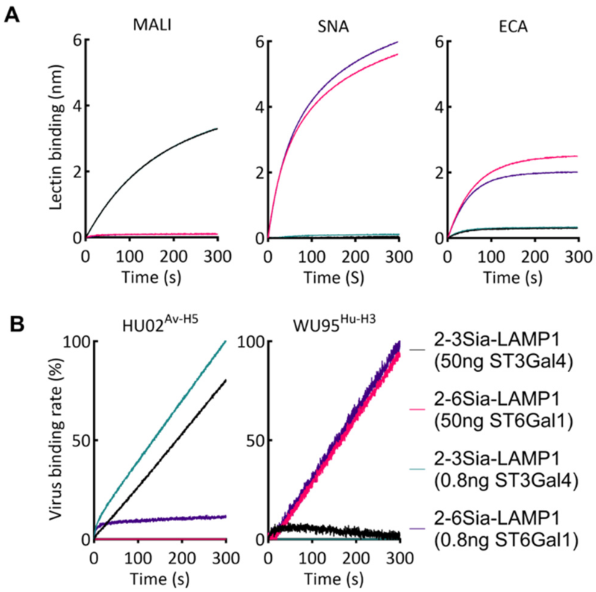

Figure S3 Analysis of lectin and virus binding to sialoglycoprotein LAMP1. Related to Figure 2. (*A*) BLI recordings of 2-3Sia-specific lectin (MALI), 2-6Sia-specific lectin (SNA) and terminal type II LN binding lectin (ECA) to LAMP1 produced in HEK$^{\Delta Sia}$ cells transfected with ST3Gal4 (2-3Sia-LAMP1) or ST6Gal1 (2-6Sia-LAMP1) as indicated. The indicated amount of STs is based on 96-well system. (*B*) BLI recordings of H5N1 strain HU02$^{Av\text{-}H5}$ and H3N2 strain WU95$^{Hu\text{-}H3}$ to 2-3Sia-LAMP1 and 2-6Sia-LAMP1 are shown.

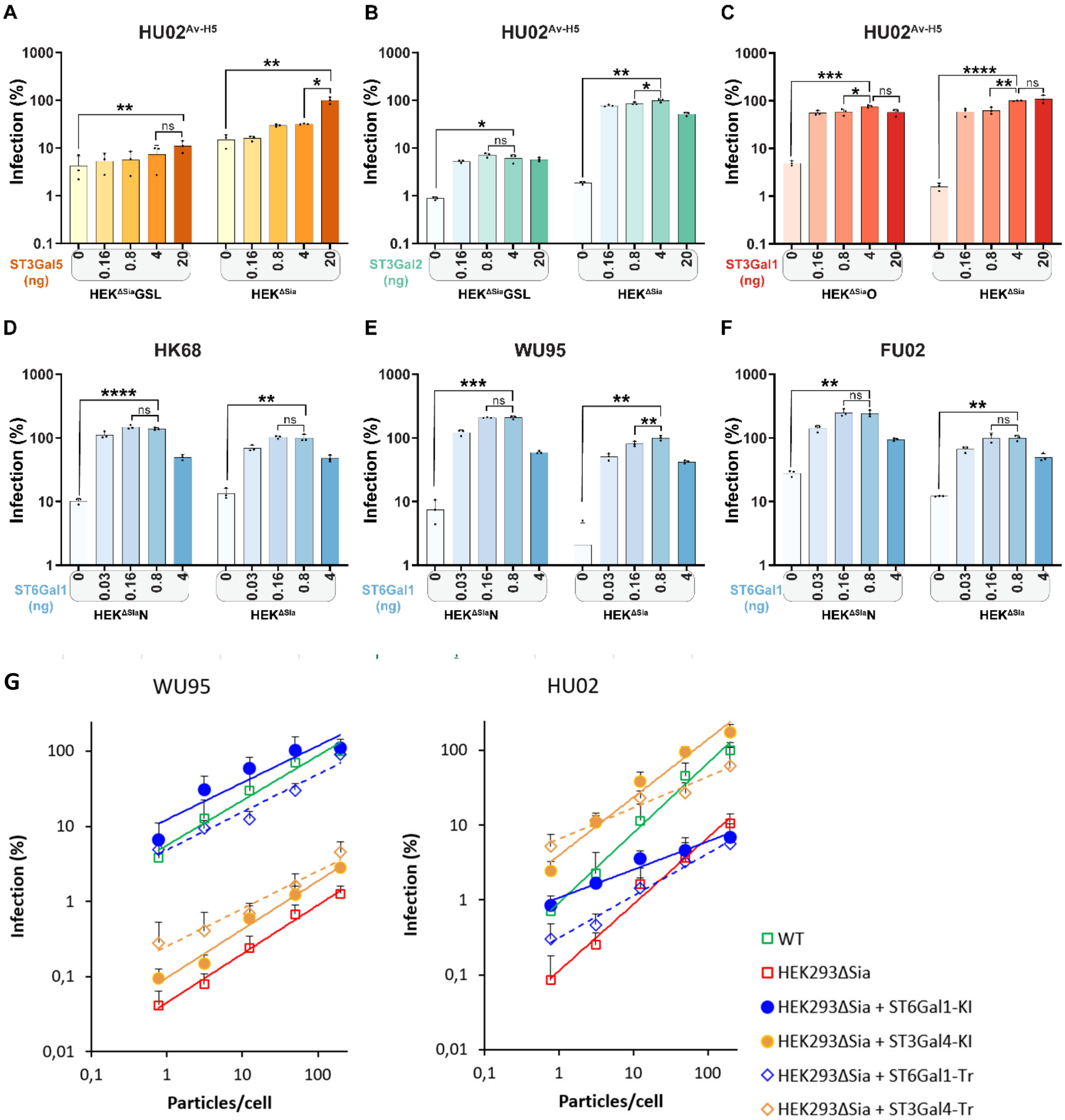

Figure S4 Infection efficiency into HEK$^{\Delta Sia}$ cells upon transient expression or stable knock-in of individual sialyltransferases. Related to Figure 3. (*A-F*) Infection efficiency of the indicated IAV strains into HEK$^{\Delta Sia}$ derived KO cell lines transfected with a serial dilution range (ng/well in 96-well plates) of the indicated sialyltransferases. Infection efficiency (Gaussia luciferase activity) at a virus dose of 100 particles/cell was determined (Y-axis; relative luciferase activity). Values are normalized to the highest value obtained in HEK$^{\Delta Sia}$ cells for each panel. (*G*). Infection efficiency of strains WU95$^{Hu\text{-}H3}$ or HU02$^{Av\text{-}H1}$ in HEK$^{\Delta Sia}$ cells transiently transfected with ST6Gal1 or ST3Gal4 (Tr; dotted lines) or carrying stable knocked-in ST6Gal1 or ST3Gal4 genes (KI).

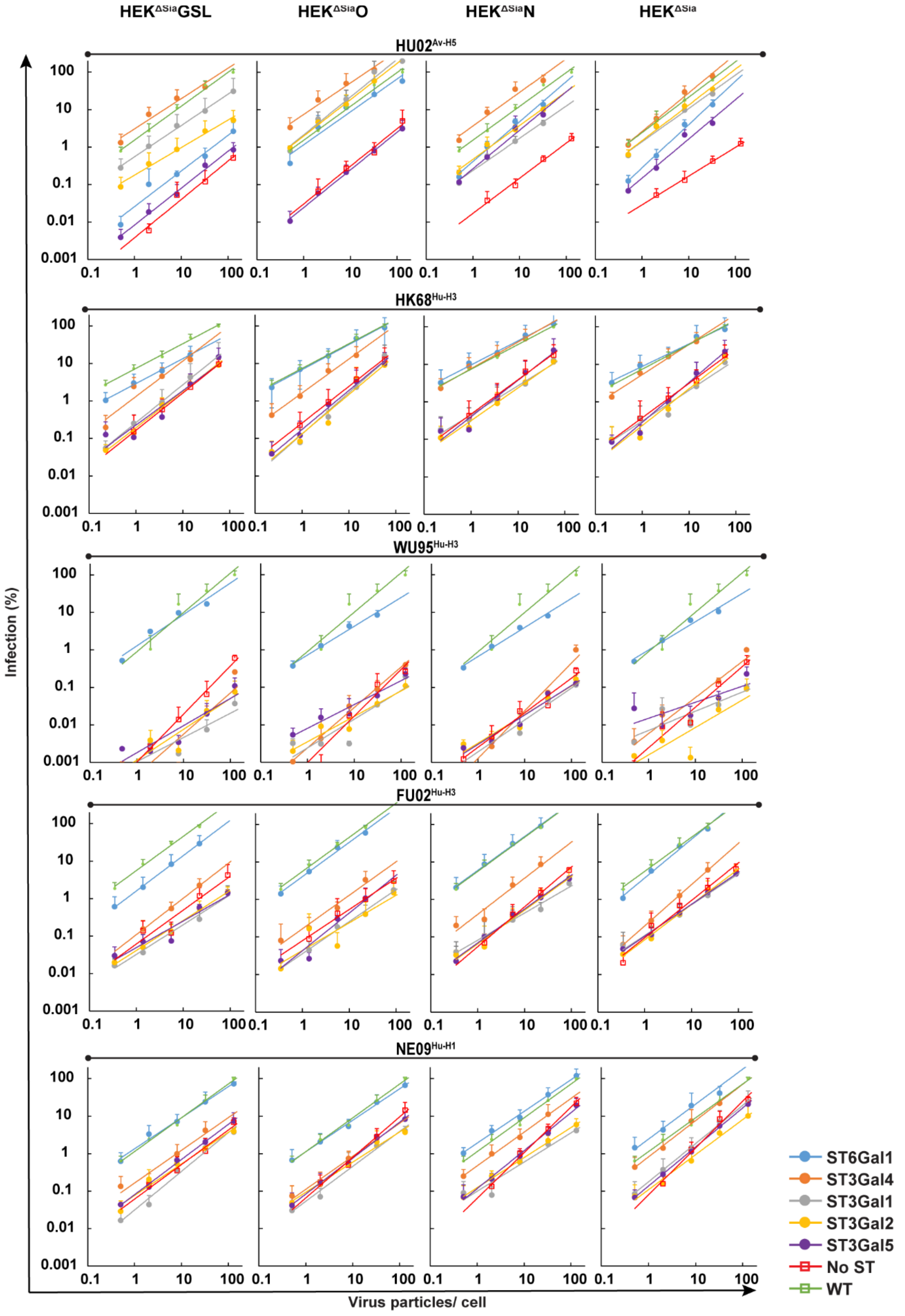

Figure S5 Determination of sialoglycotope-specific infection efficiency of avian and human IAV strains. Related to Figure 3. Infection efficiency of avian H5N1 (HU02$^{Av\text{-}H5}$), human H3N2 (HK68, WU95 and FU02), human H1N1 (NE09) in glycoconjugate-specific cell lines transfected with the indicated STs. Infection by serially diluted virus doses (x-axis, virus particles/cell) was determined by induction of Gaussia luciferase activity. Values are normalized to maximum infection levels obtained in HEK^{WT} cells

(y-axis) and curves fitted to equation $y=ax^b$ are displayed (curve fitting statistics in Table S2). HU02 and WU95 are shown in Figure 3A but included here too to enable direct comparison between strains.

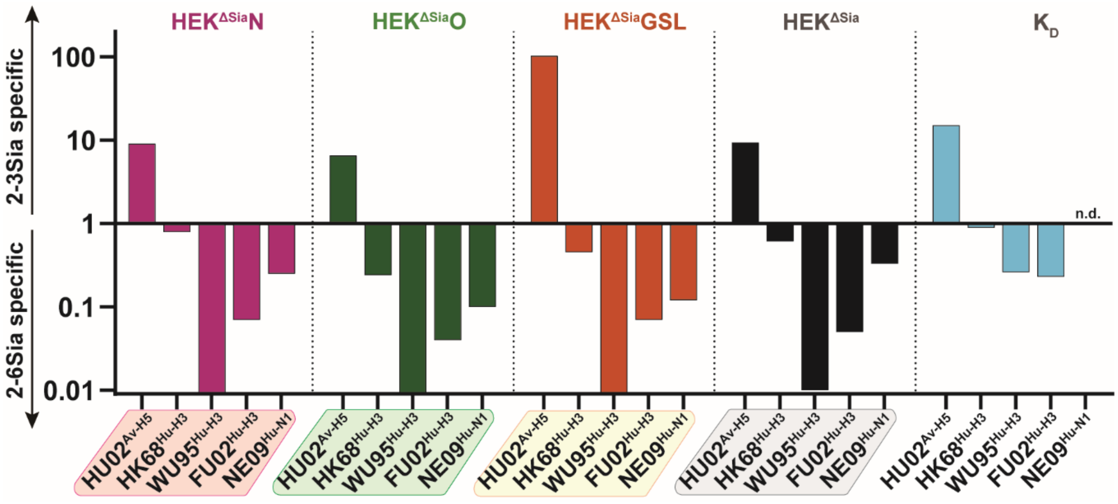

Figure S6 Analysis of IAV infection selectivity into different glycoconjugates groups. Related to Figure 3. Infection selectivity was defined as “infection into cells transfected with ST3Gal4 / infection cells transfected with ST6Gal1” and compared with binding selectivity (K_D for 2-6Sialyllactosamine / K_D for 2-3Sialyllactosamine) as previously determined (13). Specific cell lines and virus strains are indicated in the figure.

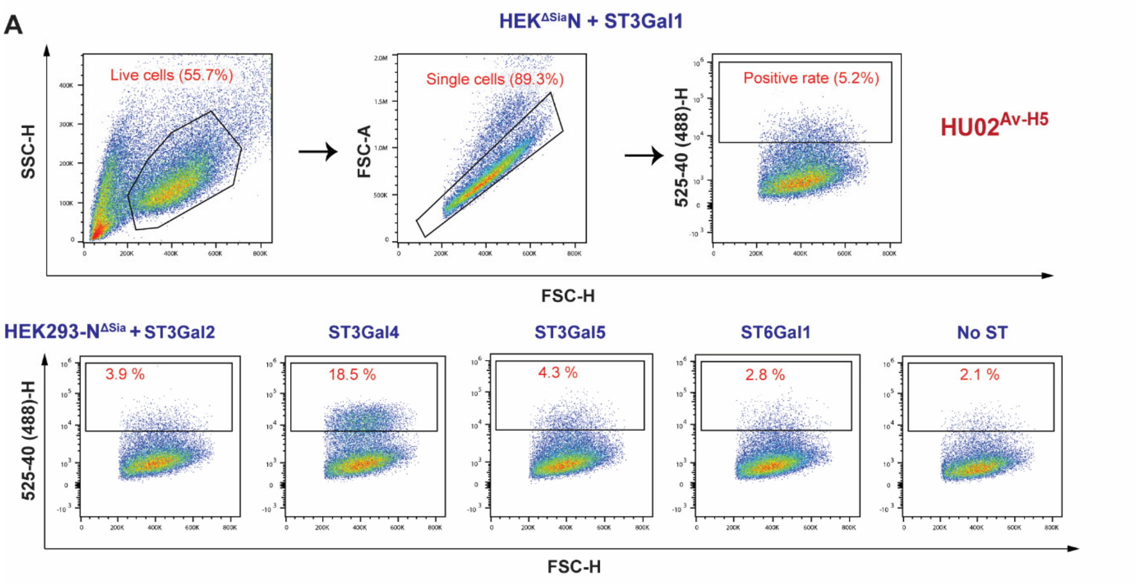

B

	ST3Gal1	ST3Gal2	ST3Gal4	ST3Gal5	ST6Gal1	No ST
Mean (525-40 (488)-H)	5.2	3.9	18.5	4.3	2.8	2.1
Positive rate (%)	2171.0	1994.0	4339.0	2035.0	1633.0	1310.0
Total fluorescence	11224.1	7796.5	80271.5	8750.5	4556.1	2751.0

Figure S7 Gating strategy of avian H5N1 HU02 binding to Sialoglycotopes. Related to Figure 4. (*A*) Representative flow cytometry gating demonstration of HU02^{Av-H5} binding to HEK$^{\Delta sia}$N cells transfected with indicated STs. (*B*) Total fluorescence of the positive-gated groups was determined and used for radar plots in Figure 4.

Table S1. Statistical analysis of curves fitted to the equation $y=ax^b$. Related to Figure 1

			a					b				
		R^2	a	SE	p	lower 95%	upper 95%	b	SE	p	lower 95%	upper 95%
**HEK^{WT}**	HK68	0.92	0.4168	0.0408	3.27E-05	0.33	0.52	0.92	0.04	4.41E-08	0.84	1.01
	EN72	0.98	0.6746	0.0580	3.08E-03	0.55	0.83	0.74	0.03	5.90E-07	0.66	0.83
	VI75	0.99	2.0961	0.0850	3.39E-04	1.85	2.38	0.65	0.04	4.34E-04	0.53	0.77
	TX77	1.00	0.2543	0.0134	1.84E-07	0.22	0.29	0.89	0.02	2.89E-08	0.83	0.95
	BK79	0.97	0.2920	0.0334	2.76E-05	0.22	0.38	0.91	0.04	8.51E-07	0.80	1.02
	SI87	0.91	0.0769	0.0080	3.26E-08	0.06	0.10	0.89	0.03	2.94E-08	0.81	0.97
	BE92	0.88	0.3601	0.0468	6.86E-05	0.27	0.48	0.89	0.05	5.49E-07	0.77	1.01
	WU95	0.91	0.1411	0.0214	2.43E-06	0.10	0.20	0.93	0.05	1.78E-07	0.82	1.03
	SY97	0.99	0.1064	0.0086	5.92E-11	0.09	0.13	1.02	0.03	1.34E-11	0.95	1.09
	FU02	0.98	0.6887	0.0871	2.03E-02	0.51	0.92	0.88	0.06	4.97E-06	0.74	1.02
	PE09	1.00	0.4260	0.0166	9.15E-08	0.39	0.47	1.01	0.02	1.38E-10	0.96	1.05
**$HEK^{\Delta Sia}$**	HK68	1.00	0.0009	0.0001	4.33E-11	0.00	0.00	1.19	0.04	1.63E-08	1.09	1.29
	EN72	0.96	0.0005	0.0001	2.63E-09	0.00	0.00	1.05	0.06	1.94E-06	0.90	1.19
	VI75	1.00	0.0012	0.0001	3.98E-10	0.00	0.00	0.86	0.04	9.67E-07	0.75	0.96
	TX77	0.89	0.0009	0.0005	3.92E-06	0.00	0.00	0.63	0.21	2.52E-02	0.11	1.15
	BK79	0.99	0.0007	0.0001	7.59E-10	0.00	0.00	0.87	0.06	1.27E-06	0.74	1.01
	SI87	0.99	0.0002	0.0001	1.82E-08	0.00	0.00	0.89	0.10	5.46E-05	0.65	1.13
	BE92	0.98	0.0002	0.0001	1.38E-08	0.00	0.00	1.09	0.12	3.82E-05	0.81	1.38
	WU95	0.98	0.0005	0.0003	4.75E-07	0.00	0.00	0.92	0.14	3.05E-04	0.59	1.25
	SY97	0.95	0.0032	0.0006	6.17E-12	0.00	0.00	0.92	0.06	3.83E-08	0.78	1.06
	FU02	0.99	0.0127	0.0011	3.92E-09	0.01	0.02	0.82	0.04	1.10E-06	0.72	0.92
	PE09	0.78	0.0203	0.0063	1.80E-06	0.01	0.04	1.08	0.12	5.56E-05	0.78	1.37
**HEK^{N}**	HK68	0.98	0.0471	0.0040	2.53E-09	0.04	0.06	0.94	0.03	1.55E-08	0.86	1.02
	EN72	0.99	0.5548	0.0692	2.43E-03	0.42	0.74	0.79	0.05	3.37E-06	0.67	0.91
	VI75	0.96	1.5508	0.1043	6.66E-03	1.26	1.91	0.87	0.06	7.77E-04	0.67	1.07
	TX77	0.97	0.2486	0.0420	1.11E-04	0.17	0.36	1.12	0.07	5.71E-06	0.93	1.30
	BK79	0.97	0.2181	0.0470	1.07E-04	0.14	0.35	0.97	0.07	2.08E-06	0.81	1.13
	SI87	0.96	0.0883	0.0071	8.50E-09	0.07	0.11	1.01	0.03	2.29E-09	0.94	1.07
	BE92	0.98	0.3033	0.0396	2.56E-05	0.23	0.41	0.99	0.05	2.61E-07	0.87	1.11
	WU95	0.98	0.1942	0.0323	1.40E-05	0.14	0.28	1.03	0.05	1.57E-07	0.91	1.14
	SY97	0.97	0.0619	0.0105	7.07E-09	0.04	0.09	1.24	0.06	2.07E-09	1.10	1.37
	FU02	0.96	1.6748	0.1700	1.77E-03	1.32	2.12	0.90	0.05	1.23E-06	0.79	1.02
	PE09	1.00	0.1062	0.0087	1.64E-08	0.09	0.13	1.09	0.04	1.16E-08	1.01	1.18
**HEK^{O}**	HK68	0.99	0.0138	0.0010	6.56E-11	0.01	0.02	1.12	0.03	1.28E-09	1.05	1.18
	EN72	1.00	0.0243	0.0024	1.70E-08	0.02	0.03	1.25	0.04	5.77E-08	1.15	1.34
	VI75	0.99	0.0352	0.0041	8.93E-08	0.03	0.05	1.09	0.05	7.99E-07	0.96	1.22
	TX77	0.98	0.0026	0.0007	2.06E-07	0.00	0.00	0.88	0.11	1.93E-04	0.61	1.14
	BK79	0.98	0.0088	0.0021	1.06E-07	0.01	0.01	0.94	0.08	5.30E-06	0.76	1.12
	SI87	0.98	0.0008	0.0004	2.69E-07	0.00	0.00	1.00	0.13	1.06E-04	0.70	1.31
	BE92	0.97	0.0049	0.0013	6.74E-08	0.00	0.01	1.04	0.10	1.30E-05	0.81	1.26
	WU95	0.98	0.0007	0.0002	1.74E-08	0.00	0.00	1.24	0.08	1.36E-06	1.05	1.44
	SY97	0.99	0.0111	0.0013	2.78E-12	0.01	0.01	1.13	0.05	2.31E-10	1.03	1.23
	FU02	0.99	0.0823	0.0053	1.66E-08	0.07	0.10	1.04	0.03	3.92E-08	0.97	1.12
	PE09	0.99	0.0224	0.0041	8.66E-08	0.02	0.03	1.13	0.08	1.75E-06	0.95	1.32
**HKE^{GSL}**	HK68	0.99	0.0414	0.0053	2.87E-08	0.03	0.06	0.89	0.05	3.43E-07	0.77	1.00
	EN72	0.95	0.1501	0.0234	1.23E-05	0.11	0.21	0.75	0.06	1.57E-05	0.60	0.89
	VI75	0.99	0.4155	0.0137	1.10E-04	0.37	0.46	0.86	0.03	1.02E-04	0.76	0.96
	TX77	0.97	0.0755	0.0091	4.93E-07	0.06	0.10	0.91	0.05	2.99E-06	0.78	1.05
	BK79	1.00	0.0236	0.0041	6.48E-08	0.02	0.03	1.01	0.06	4.08E-07	0.88	1.14
	SI87	0.98	0.0068	0.0012	8.36E-09	0.00	0.01	0.95	0.05	4.99E-07	0.82	1.08
	BE92	0.98	0.0699	0.0078	3.98E-08	0.05	0.09	0.95	0.04	1.26E-07	0.85	1.06
	WU95	0.99	0.0401	0.0055	3.93E-08	0.03	0.05	0.89	0.04	1.14E-07	0.79	0.99
	SY97	1.00	0.0124	0.0023	1.68E-10	0.01	0.02	1.31	0.07	2.42E-09	1.16	1.46
	FU02	0.96	0.1246	0.0153	1.91E-06	0.09	0.17	1.01	0.06	1.86E-06	0.87	1.15
	PE09	0.99	0.0769	0.0049	1.19E-09	0.07	0.09	1.10	0.03	2.01E-09	1.03	1.17

Table S2. Statistical analysis of curves fitted to the equation $y=ax^b$. Related to Figure 3B

				a					b				
			R^2	a	SE	p	lower 95%	upper 95%	b	SE	p	lower 95%	upper 95%
$HU02^{Av-H5}$	$HEK^{\Delta sia}GSL$	ST6Gal1	1.00	0.0259	0.0099	1.51E-03	0.01	0.07	0.95	0.11	3.54E-03	0.59	1.31
		ST3Gal4	0.96	3.0521	0.8941	4.91E-02	1.01	9.22	0.81	0.12	2.24E-02	0.28	1.34
		ST3Gal1	1.00	0.5489	0.0496	6.16E-03	0.42	0.72	0.83	0.03	1.04E-04	0.74	0.93
		ST3Gal2	0.98	0.1777	0.0271	1.19E-03	0.11	0.28	0.73	0.05	6.62E-04	0.58	0.89
		ST3Gal5	0.98	0.0083	0.0012	5.06E-05	0.01	0.01	0.98	0.05	2.47E-04	0.83	1.13
		No ST	1.00	0.0038	0.0020	5.57E-03	0.00	0.02	1.02	0.13	1.59E-02	0.46	1.59
	$HEK^{\Delta sia}O$	ST6Gal1	0.98	1.1583	0.4743	6.97E-01	0.39	3.45	0.88	0.12	5.23E-03	0.50	1.26
		ST3Gal4	0.98	7.4435	1.7225	1.06E-02	3.04	18.23	0.84	0.10	1.37E-02	0.41	1.27
		ST3Gal1	0.93	2.1237	0.5967	5.58E-02	0.97	4.67	1.01	0.09	1.35E-03	0.73	1.28
		ST3Gal2	1.00	1.9811	0.1997	1.92E-02	1.31	2.99	0.97	0.05	2.24E-03	0.77	1.17
		ST3Gal5	1.00	0.0244	0.0022	2.94E-05	0.02	0.03	1.01	0.03	6.24E-05	0.91	1.10
		No ST	0.99	0.0307	0.0114	8.07E-03	0.01	0.12	1.00	0.10	9.55E-03	0.58	1.43
	$HEK^{\Delta sia}N$	ST6Gal1	0.98	0.4051	0.0889	4.50E-02	0.17	0.95	1.07	0.09	7.77E-03	0.66	1.48
		ST3Gal4	0.90	3.6418	1.2305	4.72E-02	1.04	12.74	0.90	0.14	2.33E-02	0.30	1.50
		ST3Gal1	1.00	0.2371	0.0420	1.26E-02	0.12	0.48	0.86	0.08	8.12E-03	0.52	1.20
		ST3Gal2	1.00	0.4665	0.0867	4.65E-02	0.22	0.97	0.90	0.08	8.06E-03	0.55	1.25
		ST3Gal5	0.95	0.2637	0.0665	2.73E-02	0.10	0.69	1.03	0.11	1.08E-02	0.56	1.49
		No ST	1.00	0.0170	0.0038	2.45E-03	0.01	0.04	0.94	0.06	4.52E-03	0.67	1.21
	$HEK^{\Delta sia}$	ST6Gal1	0.99	0.2784	0.0416	1.17E-02	0.15	0.51	1.16	0.07	3.31E-03	0.87	1.44
		ST3Gal4	0.98	2.6048	0.4959	3.16E-02	1.23	5.51	1.03	0.08	6.51E-03	0.67	1.39
		ST3Gal1	0.99	1.3718	0.3142	2.65E-01	0.56	3.33	0.89	0.10	1.20E-02	0.47	1.32
		ST3Gal2	0.99	1.4676	0.2896	1.67E-01	0.68	3.19	0.95	0.09	8.05E-03	0.58	1.32
		ST3Gal5	0.93	0.1475	0.0453	1.91E-02	0.05	0.47	1.05	0.13	1.46E-02	0.50	1.60
		No ST	1.00	0.0264	0.0053	2.56E-03	0.01	0.06	0.78	0.06	5.45E-03	0.53	1.03
	HEK^{WT}		0.97	1.5386	0.2220	4.94E-02	1.00	2.36	0.90	0.05	3.08E-04	0.75	1.05
				a					b				
			R^2	a	SE	p	lower 95%	upper 95%	b	SE	p	lower 95%	upper 95%
$HK68^{Hu-H3}$	$HEK^{\Delta sia}GSL$	ST6Gal1	1.00	2.9095	0.1413	1.97E-03	2.37	3.57	0.66	0.03	1.86E-03	0.54	0.78
		ST3Gal4	0.98	1.3125	0.5407	5.13E-01	0.30	5.79	0.95	0.21	4.48E-02	0.05	1.84
		ST3Gal1	1.00	0.2635	0.0126	9.50E-05	0.23	0.31	1.01	0.02	1.70E-05	0.95	1.07
		ST3Gal2	1.00	0.1938	0.0200	4.66E-04	0.14	0.26	0.97	0.04	1.74E-04	0.84	1.11
		ST3Gal5	0.99	0.2257	0.1139	3.56E-02	0.06	0.83	0.92	0.17	1.32E-02	0.37	1.48
		No ST	1.00	0.1656	0.0025	7.01E-05	0.16	0.18	1.00	0.01	3.61E-05	0.97	1.02
	$HEK^{\Delta sia}O$	ST6Gal1	0.98	6.8483	0.5143	1.17E-04	5.44	8.62	0.67	0.03	2.17E-04	0.57	0.77
		ST3Gal4	0.99	1.6344	0.1583	3.36E-02	1.10	2.43	0.91	0.06	3.76E-03	0.67	1.15
		ST3Gal1	1.00	0.1319	0.0381	4.09E-03	0.06	0.30	1.10	0.11	2.01E-03	0.76	1.45
		ST3Gal2	0.95	0.1335	0.0522	8.84E-03	0.05	0.38	1.05	0.14	5.03E-03	0.60	1.50
		ST3Gal5	0.99	0.1748	0.0241	8.74E-04	0.12	0.26	1.04	0.06	3.22E-04	0.87	1.22
		No ST	1.00	0.2594	0.0152	1.78E-03	0.20	0.33	0.98	0.02	5.38E-04	0.88	1.08
	$HEK^{\Delta sia}N$	ST6Gal1	0.99	9.6370	0.8338	1.08E-04	7.40	12.55	0.64	0.04	3.64E-04	0.53	0.76
		ST3Gal4	1.00	7.6335	0.8218	2.52E-03	4.92	11.85	0.71	0.06	7.44E-03	0.45	0.98
		ST3Gal1	0.99	0.4470	0.0626	8.69E-03	0.29	0.68	0.77	0.06	8.17E-04	0.60	0.95
		ST3Gal2	1.00	0.3128	0.0435	2.97E-03	0.21	0.47	0.87	0.06	5.65E-04	0.69	1.05
		ST3Gal5	1.00	0.4030	0.1333	5.00E-02	0.16	1.00	0.97	0.12	4.15E-03	0.58	1.36
		No ST	0.99	0.4687	0.0635	2.70E-02	0.27	0.81	0.92	0.05	3.03E-03	0.70	1.13
	$HEK^{\Delta sia}$	ST6Gal1	0.95	8.6857	1.0227	2.98E-04	6.09	12.38	0.60	0.05	1.06E-03	0.45	0.75
		ST3Gal4	0.99	5.2846	0.6068	4.24E-03	3.31	8.44	0.81	0.07	6.53E-03	0.52	1.09
		ST3Gal1	0.99	0.3033	0.0824	1.57E-02	0.14	0.65	0.82	0.10	4.09E-03	0.49	1.15
		ST3Gal2	0.99	0.2299	0.0792	1.57E-02	0.09	0.59	0.98	0.13	4.41E-03	0.58	1.39
		ST3Gal5	1.00	0.2801	0.0682	1.00E-02	0.14	0.56	1.07	0.09	1.43E-03	0.77	1.36
		No ST	0.99	0.3759	0.0484	1.50E-02	0.22	0.63	0.92	0.05	2.73E-03	0.71	1.13
	HEK^{WT}		0.99	7.3781	0.4864	7.16E-05	6.02	9.04	0.66	0.03	1.55E-04	0.57	0.75

WU95^{Hu-H3}

			a					b				
		R^2	a	SE	p	lower 95%	upper 95%	b	SE	p	lower 95%	upper 95%
HEK$^{\Delta sia}$GSL	ST6Gal1	0.92	1.2810	0.4361	4.87E-01	0.36	4.52	0.84	0.14	2.75E-02	0.23	1.45
	ST3Gal4	0.98	0.0005	0.0007	3.96E-03	0.00	0.01	1.10	0.34	4.67E-02	0.03	2.16
	ST3Gal1	0.95	0.0010	0.0014	1.48E-02	0.00	0.04	0.65	0.27	1.38E-01	-0.51	1.80
	ST3Gal2	0.99	0.0011	0.0018	1.92E-02	0.00	0.07	0.82	0.30	1.13E-01	-0.48	2.12
	ST3Gal5	0.96	0.0018	0.0012	1.11E-03	0.00	0.01	0.72	0.18	2.70E-02	0.16	1.29
	No ST	1.00	0.0010	0.0003	1.23E-03	0.00	0.00	1.28	0.08	3.56E-03	0.95	1.61
HEK$^{\Delta sia}$O	ST6Gal1	0.97	0.7222	0.1119	1.52E-01	0.39	1.34	0.77	0.07	8.20E-03	0.47	1.07
	ST3Gal4	1.00	0.0024	0.0004	3.37E-05	0.00	0.00	1.08	0.05	2.56E-04	0.91	1.24
	ST3Gal1	0.97	0.0024	0.0022	2.58E-03	0.00	0.02	0.76	0.23	4.33E-02	0.04	1.48
	ST3Gal2	0.99	0.0035	0.0012	3.29E-04	0.00	0.01	0.68	0.11	7.74E-03	0.34	1.01
	ST3Gal5	0.97	0.0081	0.0019	1.94E-04	0.00	0.02	0.63	0.08	3.56E-03	0.39	0.88
	No ST	0.93	0.0010	0.0005	5.51E-04	0.00	0.00	1.24	0.15	4.03E-03	0.75	1.73
HEK$^{\Delta sia}$N	ST6Gal1	0.97	0.6670	0.1041	1.08E-01	0.36	1.25	0.78	0.07	8.13E-03	0.47	1.08
	ST3Gal4	0.99	0.0013	0.0005	3.33E-04	0.00	0.00	1.27	0.13	2.04E-03	0.87	1.67
	ST3Gal1	0.98	0.0019	0.0013	6.80E-03	0.00	0.02	0.85	0.16	3.54E-02	0.14	1.56
	ST3Gal2	1.00	0.0032	0.0013	4.46E-04	0.00	0.01	0.76	0.12	7.66E-03	0.39	1.14
	ST3Gal5	0.95	0.0030	0.0010	2.63E-04	0.00	0.01	0.79	0.10	4.41E-03	0.46	1.11
	No ST	0.97	0.0025	0.0008	2.28E-04	0.00	0.01	0.92	0.10	2.64E-03	0.61	1.24
HEK$^{\Delta sia}$	ST6Gal1	0.94	0.9914	0.2069	9.68E-01	0.44	2.24	0.75	0.09	1.46E-02	0.36	1.15
	ST3Gal4	0.99	0.0058	0.0041	2.40E-03	0.00	0.03	0.96	0.19	1.48E-02	0.36	1.56
	ST3Gal1	0.90	0.0070	0.0044	2.01E-03	0.00	0.03	0.51	0.17	5.85E-02	-0.03	1.06
	ST3Gal2	0.98	0.0016	0.0016	2.67E-03	0.00	0.01	0.73	0.25	5.89E-02	-0.05	1.51
	ST3Gal5	0.86	0.0149	0.0115	5.24E-03	0.00	0.09	0.43	0.20	1.25E-01	-0.22	1.07
	No ST	1.00	0.0024	0.0010	4.02E-04	0.00	0.01	1.08	0.12	2.94E-03	0.70	1.46
HEK^{WT}		0.98	0.8939	0.9568	8.92E-01	0.04	20.47	1.05	0.23	4.52E-02	0.06	2.04

FU02^{Hu-H3}

			a					b				
		R^2	a	SE	p	lower 95%	upper 95%	b	SE	p	lower 95%	upper 95%
HEK$^{\Delta sia}$GSL	ST6Gal1	1.00	1.6013	0.0605	6.15E-03	1.37	1.88	0.94	0.02	4.50E-04	0.86	1.03
	ST3Gal4	1.00	0.1097	0.0082	2.07E-02	0.04	0.27	0.97	0.03	2.29E-02	0.53	1.41
	ST3Gal1	0.98	0.0322	0.0053	1.90E-04	0.02	0.05	0.80	0.06	8.52E-04	0.61	0.98
	ST3Gal2	1.00	0.0413	0.0040	5.24E-05	0.03	0.06	0.80	0.04	1.88E-04	0.69	0.91
	ST3Gal5	0.99	0.0496	0.0184	2.45E-03	0.02	0.14	0.71	0.12	9.88E-03	0.32	1.09
	No ST	1.00	0.0616	0.0603	5.50E-02	0.00	1.16	0.90	0.24	6.31E-02	-0.12	1.93
HEK$^{\Delta sia}$O	ST6Gal1	0.98	4.0660	0.5059	6.92E-03	2.45	6.73	0.91	0.06	4.73E-03	0.64	1.19
	ST3Gal4	0.99	0.1652	0.0370	1.23E-02	0.07	0.39	0.89	0.11	1.45E-02	0.43	1.36
	ST3Gal1	0.99	0.0355	0.0076	3.38E-03	0.02	0.08	0.86	0.07	6.26E-03	0.56	1.15
	ST3Gal2	0.97	0.0434	0.0300	9.41E-03	0.01	0.23	0.74	0.20	3.54E-02	0.10	1.38
	ST3Gal5	1.00	0.0425	0.0216	1.65E-02	0.01	0.25	1.01	0.22	4.50E-02	0.06	1.96
	No ST	1.00	0.0789	0.0177	6.31E-03	0.03	0.19	0.83	0.07	7.13E-03	0.53	1.14
HEK$^{\Delta sia}$N	ST6Gal1	1.00	5.9512	0.4891	1.95E-03	4.24	8.36	0.91	0.04	2.20E-03	0.72	1.09
	ST3Gal4	1.00	0.3963	0.1447	9.69E-02	0.10	1.51	0.96	0.17	2.89E-02	0.24	1.69
	ST3Gal1	0.97	0.0810	0.0126	4.17E-04	0.05	0.13	0.72	0.06	9.74E-04	0.55	0.90
	ST3Gal2	1.00	0.0654	0.0139	7.58E-04	0.04	0.12	0.90	0.07	1.19E-03	0.67	1.13
	ST3Gal5	1.00	0.0692	0.0071	1.08E-04	0.05	0.09	0.90	0.04	1.57E-04	0.78	1.02
	No ST	1.00	0.0547	0.0087	2.57E-03	0.03	0.10	1.06	0.05	2.34E-03	0.84	1.29
HEK$^{\Delta sia}$	ST6Gal1	0.99	3.6862	0.5117	9.78E-03	2.11	6.45	1.03	0.07	4.55E-03	0.73	1.34
	ST3Gal4	1.00	0.1960	0.0052	2.56E-04	0.18	0.22	1.10	0.01	1.62E-04	1.04	1.16
	ST3Gal1	0.99	0.1083	0.0193	8.65E-04	0.06	0.18	0.82	0.06	9.65E-04	0.62	1.02
	ST3Gal2	1.00	0.0830	0.0088	1.46E-04	0.06	0.11	0.94	0.04	1.51E-04	0.82	1.07
	ST3Gal5	1.00	0.1039	0.0082	8.40E-05	0.08	0.13	0.85	0.03	8.90E-05	0.76	0.95
	No ST	0.99	0.1571	0.0078	6.82E-04	0.13	0.19	0.83	0.02	4.11E-04	0.76	0.90
HEK^{WT}		0.99	5.5400	0.6122	3.73E-03	3.53	8.70	0.91	0.06	3.82E-03	0.67	1.15

NE09$^{Hu\text{-}H1}$

			a					b				
		R^2	a	SE	p	lower 95%	upper 95%	b	SE	p	lower 95%	upper 95%
HEK$^{\Delta sia}$GSL	ST6Gal1	1.00	1.3396	0.2083	1.36E-01	0.85	2.12	0.83	0.05	4.86E-04	0.67	0.99
	ST3Gal4	0.99	0.1631	0.0683	3.52E-02	0.04	0.73	0.87	0.17	3.49E-02	0.15	1.59
	ST3Gal1	0.98	0.0311	0.0079	6.03E-04	0.02	0.06	1.03	0.08	9.62E-04	0.78	1.28
	ST3Gal2	1.00	0.0756	0.0194	1.48E-03	0.04	0.16	0.84	0.08	1.80E-03	0.59	1.10
	ST3Gal5	1.00	0.0794	0.0079	1.15E-04	0.06	0.11	0.94	0.03	9.55E-05	0.83	1.04
	No ST	0.99	0.0609	0.0239	1.37E-02	0.01	0.25	0.92	0.10	1.25E-02	0.47	1.37
HEK$^{\Delta sia}$O	ST6Gal1	1.00	1.1213	0.1018	2.79E-01	0.85	1.48	0.84	0.03	1.03E-04	0.74	0.93
	ST3Gal4	1.00	0.1116	0.0233	7.39E-03	0.05	0.25	0.88	0.09	1.04E-02	0.49	1.27
	ST3Gal1	0.98	0.0541	0.0145	1.16E-03	0.03	0.12	0.93	0.08	1.51E-03	0.67	1.19
	ST3Gal2	0.95	0.0934	0.0150	5.36E-04	0.06	0.15	0.80	0.05	5.81E-04	0.64	0.97
	ST3Gal5	1.00	0.0832	0.0059	4.52E-05	0.07	0.10	0.96	0.02	3.34E-05	0.88	1.03
	No ST	1.00	0.0663	0.0186	8.18E-03	0.02	0.19	1.08	0.08	5.09E-03	0.75	1.41
HEK$^{\Delta sia}$N	ST6Gal1	1.00	1.8943	0.1909	6.92E-03	1.40	2.57	0.85	0.03	1.36E-04	0.74	0.95
	ST3Gal4	1.00	0.4697	0.0389	1.09E-02	0.33	0.66	0.90	0.04	1.79E-03	0.74	1.06
	ST3Gal1	0.99	0.0990	0.0418	7.18E-03	0.03	0.30	0.78	0.12	7.91E-03	0.39	1.17
	ST3Gal2	1.00	0.1245	0.0109	1.44E-04	0.10	0.16	0.80	0.03	1.07E-04	0.71	0.89
	ST3Gal5	1.00	0.1164	0.0162	4.81E-04	0.08	0.18	1.01	0.05	1.96E-04	0.87	1.16
	No ST	1.00	0.0647	0.0140	5.05E-03	0.03	0.15	1.22	0.06	2.52E-03	0.95	1.48
HEK$^{\Delta sia}$	ST6Gal1	0.98	2.4364	0.5461	2.17E-02	1.28	4.64	0.93	0.07	9.31E-04	0.71	1.16
	ST3Gal4	0.99	0.8131	0.0992	2.14E-01	0.50	1.33	0.97	0.06	3.22E-03	0.73	1.21
	ST3Gal1	0.99	0.1768	0.0133	1.61E-04	0.14	0.22	1.02	0.03	3.36E-05	0.94	1.10
	ST3Gal2	0.99	0.1088	0.0209	1.08E-03	0.06	0.19	0.94	0.06	6.13E-04	0.74	1.13
	ST3Gal5	1.00	0.1359	0.0053	1.57E-05	0.12	0.15	1.04	0.01	4.68E-06	1.00	1.08
	No ST	0.98	0.0796	0.0320	1.74E-02	0.02	0.34	1.25	0.11	7.11E-03	0.79	1.71
HEK^{WT}		1.00	1.1271	0.1323	3.60E-01	0.79	1.60	0.90	0.04	1.73E-04	0.78	1.02
